# Thyroid hormone receptor beta signaling is a targetable driver of prostate cancer growth

**DOI:** 10.1186/s12943-025-02451-2

**Published:** 2025-10-14

**Authors:** Aleksandra Fesiuk, Daniel Pölöske, Elvin D. de Araujo, Geordon A. Frere, Timothy B. Wright, Gary Tin, Yasir S. Raouf, Olasunkanmi O. Olaoye, Ji Sung Park, Nicolas Blavet, Boris Tichý, Michaela Schlederer, Sandra Högler, Michael Wolf, Cécile Philippe, Osman Aksoy, Adam Varady, Alejandro Medaglia Mata, Maxim Varenicja, Boglárka Szabó, Theresa Weiss, Gabriel Wasinger, Torben Redmer, Heidi A. Neubauer, Martin Susani, Clemens P. Spielvogel, Jing Ning, Maik Dahlhoff, Martin Schepelmann, Richard Kennedy, Richard Moriggl, Geoffrey Brown, Jenny Persson, Christopher Gerner, Vojtech Bystry, Oldamur Hollóczki, David M. Heery, Patrick T. Gunning, Olaf Merkel, Brigitte Hantusch#, Lukas Kenner

**Affiliations:** 1 Department of Pathology, Medical University of Vienna, Vienna, Austria; 2 Christian Doppler Laboratory for Applied Metabolomics, Division of Nuclear Medicine, The Department of Biomedical Imaging and Image-Guided Therapy, Medical University of Vienna, Vienna, Austria; 3 Department of Biomedical Imaging and Image Guided Therapy, Division of Nuclear Medicine, Medical University Vienna, Vienna, Austria; 4 Unit of Laboratory Animal Pathology, Department of Biological Sciences and Pathobiology, University of Veterinary Medicine Vienna, Vienna, Austria; 5 Department of Chemical and Physical Sciences, University of Toronto Mississauga, 3359 Mississauga Road, Mississauga, ON L5L 1C6 Canada; 6 Department of Chemistry, University of Toronto, 80 St. George Street, Toronto, ON M5S 3H6 Canada; 7 Centre for Biological Sciences, University of Veterinary Medicine, Vienna, Austria; 8 St. Anna Children’s Cancer Research Institute, Innovative Cancer Models, Vienna, Austria; 9 Central European Institute of Technology, Masaryk University, Brno, 62500 Czech Republic; 10 Department of Physical Chemistry, University of Debrecen, Egyetem tér 1, Debrecen, 4032 Hungary; 11 Institute of in vivo and in vitro Models, Department of Biological Sciences and Pathobiology, University of Veterinary Medicine Vienna, Veterinärplatz 1, Vienna, 1210 Austria; 12 Institute for Pathophysiology and Allergy Research, Medical University of Vienna, Waehringer Guertel 18-20, Vienna, 1090 Austria; 13 Centre for Cancer Research and Cell Biology, Almac Diagnostics, Queens University Belfast, Northern Ireland, Northern Ireland, Craigavon United Kingdom; 14 School of Biomedical Sciences, Institute of Clinical Sciences, College of Medical and Dental Sciences, University of Birmingham, Birmingham, UK; 15 Division of Experimental Cancer Research, Department of Translational Medicine, Clinical Research Centre, Lund University, Malmö, Sweden; 16 Department of Molecular Biology, Umeå University, Umeå, Sweden; 17 Division of Biomolecular Science and Medicinal Chemistry, School of Pharmacy, BioDiscovery Institute, University of Nottingham, Nottingham, UK; 18 European Research Initiative on ALK-Related Malignancies (ERIA), Cambridge, UK; 19 Center for Biomarker Research in Medicine (CBmed), Graz, Austria; 20 Comprehensive Cancer Center, Medical University Vienna, Vienna, Austria; 21 Department for Basic and Translational Oncology and Hematology, Division Molecular Oncology and Hematology, Karl Landsteiner University of Health Sciences, Krems, 3500 Austria; 22 Department of Analytical Chemistry, University of Vienna, Vienna, 1090 Austria; 23 Doctoral School in Chemistry, University of Vienna, Vienna, 1090 Austria; 24 Joint Metabolome Facility, University of Vienna, Vienna, 1090 Austria; 25 Department of Biosciences & Medical Biology, Paris Lodron University of Salzburg, Salzburg, Austria

**Keywords:** Thyroid hormone receptor β, Prostate cancer, NH-3, Androgen receptor, Murine PCa model

## Abstract

Thyroid hormone (TH) signaling plays a major role in the development, energy homeostasis, and metabolism of most tissues. Recent studies have identified THs as drivers of prostate cancer (PCa) development and progression. We reported that the T3-scavenger protein µ-crystallin (CRYM) regulates the development and progression of PCa and that this involved crosstalk with androgen receptor (AR) signaling. However, the mechanisms remain incompletely understood. Here, we explored the role of thyroid hormone receptor β (TRβ), which is the main effector of TH signaling, in the context of PCa. The use of the TRβ-selective antagonist NH-3 inhibited PCa cell proliferation in vitro and reduced tumor size in PCa xenograft models in vivo. Notably, NH-3 was highly effective in the engrafted 22Rv1 cell line, a model for castration-resistant PCa (CRPC). Mechanistic studies revealed that NH-3 downregulates AR and the AR target genes *Nkx3.1* and *KLK3* (*PSA*). NH-3 was a more effective anticancer agent than enzalutamide, and their combined use was synergistic. Evidence from human datasets corroborates our findings, whereby elevated TRβ expression and mutations in the TH signaling pathway are associated with the onset of PCa. Collectively, these results establish TRβ as a mediator of tumorigenesis in PCa and identify NH-3 as a promising therapeutic agent for targeting AR signaling, particularly in CRPC.

**Graphical Abstract:**

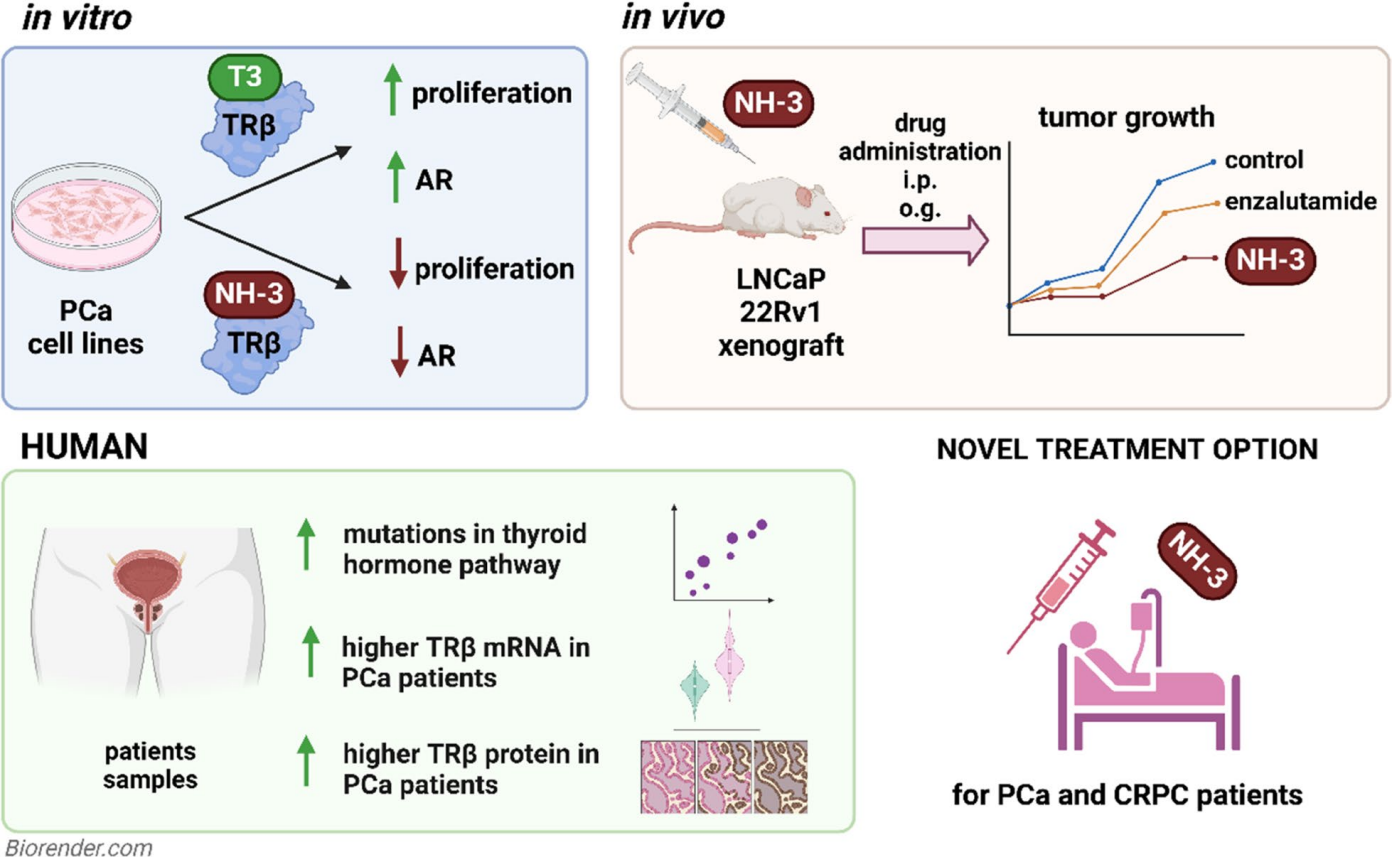

**Supplementary Information:**

The online version contains supplementary material available at 10.1186/s12943-025-02451-2.

## Background

Prostate cancer (PCa) is the second most frequent malignancy in men [1] and the sixth leading cause of death worldwide [2]. Epidemiological studies estimate that one in eight men will be diagnosed with PCa during their lifetime [1]. Disease progression highly depends on androgen receptor (AR) signaling, which regulates cell proliferation and survival. The AR, a nuclear receptor and ligand-dependent transcription factor, is activated by testosterone and dihydrotestosterone (DHT). Given its central role in PCa growth, most therapeutic strategies target AR signaling. The first-line treatment of PCa is androgen deprivation therapy (ADT), which suppresses testosterone and DHT through either surgical castration or anti-androgen drugs [3]. Second-generation AR inhibitors, such as enzalutamide, further block AR activity [4]. However, up to 20% of patients develop resistance to ADT and progress to castration-resistant prostate cancer (CRPC) [5]. Nearly 50% of CRPC cases advance to metastatic CRPC (mCRPC) within three years of diagnosis [6], resulting in a poor prognosis and a diminished quality of life.

Recent studies have shown that THs regulate the pituitary-gonadal axis and androgen signaling [7] and have linked altered serum levels of THs and thyroid-stimulating hormone (TSH) with the risk of PCa [8–10]. At the cellular level, THs promote PCa cell proliferation in vitro [7, 11, 12] and tumor progression in vivo [13]. TH signaling has been implicated in PCa development and progression by stimulating cell proliferation and tumor growth. However, the molecular mechanisms underlying this process remain unclear.

The recent FDA approval of the TRβ-selective agonist resmetirom for treating non-alcoholic steatohepatitis (NASH) highlights that the therapeutic potential of targeting TRβ [14, 15] is being recognized.

Our previous findings identified reduced expression of the T3-scavenger protein µ-crystallin (CRYM) in aggressive PCa, correlating with earlier biochemical recurrence and poor survival [16]. CRYM is a negative regulator of T3 availability, implying enhanced T3-mediated signaling when CRYM is downregulated [17]. CRYM overexpression reduces TRβ expression, suggesting regulatory crosstalk with AR signaling, evidenced by suppressed PSA (prostate-specific antigen, *KLK3*). Based on these findings, we hypothesize that TRβ is a key driver of PCa progression and a potential therapeutic target.

In this study, we investigated the role of TRβ in PCa proliferation and tumor development. We demonstrate that the TRβ-selective antagonist NH-3 significantly reduced PCa cell proliferation in vitro and suppressed tumor growth in xenograft models in vivo. NH-3 treatment led to AR degradation and loss of AR-dependent gene expression. Analysis of human datasets revealed enhanced *TRHB* mRNA expression and TRβ protein levels in PCa compared to normal tissue, providing further evidence for a tumor-driving role of TRβ in PCa patients. Our results show that TRβ blockade is a promising therapeutic approach, particularly for patients with ADT resistance.

## Methods

### PCa patient cohorts

#### Vienna cohort

This study utilized formalin-fixed, paraffin-embedded (FFPE) samples and data previously published by Ning J. et al.l [18].

The waterfall plot was generated using R with the GenVisR package [19].

#### Tuebingen cohort

FFPE samples were collected at the Institute of Pathology, Tuebingen, Germany, from patients who had undergone radical prostatectomy. Immunohistochemistry (IHC) was performed following standard protocols, and protein expression was quantified as previously described [20]. The following TRβ antibody was used: Cat# 209-301‐A96, 1:100, Rockland. Tissue microarrays (TMAs) were evaluated by four certified pathologists according to a 4‐point scale for staining intensity (0‐3) and a 4‐point scale for the number of stained cells (0‐3).

### Publicly available databases

*THRB* mRNA expression levels were obtained from The Cancer Genome Atlas (TCGA) from the following datasets: prostate adenocarcinoma (PRAD), bladder urothelial carcinoma (BLCA), breast cancer (BRCA), colon adenocarcinoma (COAD), glioblastoma multiforme (GBM), head and neck squamous cell carcinoma (HNSC), liver hepatocellular carcinoma (LIHC), lung adenocarcinoma (LUAD), ovarian carcinoma (OV), pancreatic adenocarcinoma (PAAD), testicular germ cell tumors (TGCT), thyroid carcinoma (THCA). For tumor compared to normal sample analysis, the GEPIA tool was used (http://gepia.cancer-pku.cn). The ENSG00000151090 identifier was used for the *THRB* gene (Ensembl version 109). Expression levels are reported as normalized Transcripts Per Million (TPM) values, which were derived from RNA-sequencing experiments conducted from tissue samples.

Gene expression data for *THRB* mRNA were extracted from various PCa transcriptomes (Grasso Prostate, Luo Prostate 2, Arredouani Prostate), including normal and tumor samples, using the Oncomine Research Premium Edition database (Thermo Fisher, Ann Arbor, MI) [21].

### Principal component analysis (PCA)

To visualize the expression levels of *THRB* in PCa samples from individual patients and healthy tissue, we examined the dataset from [22], and used the autoplot function of the R package ggplot2. Boxplot representations of *THRB* expression in PCa and healthy normal tissue of stated samples show the median (center line), the upper and lower quartiles (the box), and the range of the data (the whiskers), including outliers. Significance was determined by an unpaired, two-tailed t-test using the R package ggplot2.

### Cell culture

#### Cell lines

Human RWPE1, BPH1, LNCaP, 22Rv1, DU145 and PC-3, prostate cancer cell lines were obtained from the American Type Culture Collection (ATCC, Manassas, VA, USA) and cultured in RPMI 1640 medium supplemented with 10% fetal bovine serum (FBS) and 1% penicillin-streptomycin (Gibco, #11875093, #15140122 and #26140079, respectively). Cells were maintained in a humidified incubator at 37 °C with 5% CO_2_. All cell lines were tested to be free of mycoplasma infection.

### Resazurin assay

Cells were seeded in 96-well plates at a density of 2-3.5 × 10 3 cells per well. Cells were cultured in full media (FM: RPMI, 10% FCS, 1% PenStrep), hormone-free media (HFM: phenol red-free RPMI (Gibco, #11835030), 10% charcoal-stripped FBS (Gibco, #12676029), 1% PenStrep) and hormone-free media with DMSO (HFM + DMSO). 10 µM bortezomib (MedChemExpress, HY-10227) was used as a positive control for cell death. After respective treatment time points, Resazurin reagent (Santa Cruz, # sc-206037) was added to each well and incubated for 3 h at 37 °C. Light emission of the reduced fluorescent product resorufin was measured using a TECAN Synergy H1 plate reader (Tecan Group) at 570 nm. The fluorescence intensity was recorded, and the relative cell viability or proliferation was calculated by comparing the fluorescence signals of the treated wells to those of the control wells. Statistical analysis was performed using one-way ANOVA and significance was defined at *p* < 0.05.

### Drug treatments

The selective TRβ inhibitor NH-3 (synthesized in the laboratory of Patrick Gunning, University of Toronto), 1-850 (Sigma-Aldrich, 609315), enzalutamide (Axon, MDV 3100), bicalutamide (MedChemExpress, HY-14249; Sigma-Aldrich, B9061), and T3 (MedChemExpress, HY-A0070A) were diluted in DMSO and treatments were performed in FM or HFM as described above. Each treatment condition was performed in triplicates.

### IncuCyte cell proliferation assay

The IncuCyte^®^ S3 Live-Cell Analysis System (Sartorius, Michigan, MI, USA) was used according to the manufacturer’s protocol for real-time kinetic monitoring of cell growth upon treatments as described previously. Phase-contrast images were automatically acquired from multiple fields per well every 2 h for the duration of the experiment. Cell proliferation was quantified using the IncuCyte integrated analysis software. The software’s built-in algorithm was used to calculate cell monolayer density based on the phase-contrast images. This metric provides a measure of cell confluence and growth over time.

### Ki-67 analysis

Ki-67 staining was performed to analyze the proliferative activity of target cells. Cells were fixed with 70% ethanol and incubated at −20 °C for 1 h. After two washes with Cell Staining Buffer (#420201, BioLegend^®^), cells were resuspended and incubated with an anti-Ki-67 antibody (#350501, BioLegend^®^) for 30 min at room temperature in the dark. Following two additional washes, cells were incubated with a secondary antibody, goat anti-mouse IgG (H + L) Cross-Adsorbed Secondary Antibody, Alexa Fluor™ 488 (# A-11001, Invitrogen), for 20–30 min in the dark. Stained cells were resuspended in cell staining buffer for flow cytometric analysis. All samples were acquired with a BD FACSCanto II (Becton Dickinson, Franklin Lakes, NJ, USA) and analyzed with FlowJo 7.6 software (BD Biosciences).

### Competitive radioligand binding assay

Radioligand binding assays for TRβ (#285940), RXRα (#269500), RXRβ (#269540), AR (#933), ERα (#5484), ERβ (#226050), GR (#469), and PR (#2341) was performed at Eurofins (https://www.eurofins.com/) according to the company standards. To evaluate the capacity of NH-3 (MUV-1, PT# 1281206) to interfere with respective radiolabeled ligand binding, increasing concentrations of NH-3 were applied, and the radioactive signals were monitored. IC50 values were determined by a non-linear, least squares regression analysis using MathIQTM (ID Business Solutions Ltd., UK). Inhibition constants (Ki) values were calculated using the equation of Cheng and Prusoff (Cheng, Y., Prusoff, W.H., Biochem. Pharmacol. 22:3099–3108, 1973) using the observed IC50 of the tested compound, the concentration of radioligand employed in the assay, and values for the KD of the ligand that were obtained experimentally at Eurofins Panlabs, Inc. The Hill coefficient (nH), which defines the slope of the competitive binding curve, was calculated using MathIQTM.

### In vitro [^68^Ga]PSMA uptake

LNCaP cells were treated with NH-3 as described above, washed with PBS, trypsinized and centrifuged. 100,000 cells were incubated in 96-well filter plates (MADVN6550, Merck Millipore, Darmstadt, Germany) with 36 kBq [^68^Ga]PSMA-11 for 1 h in an incubator (humidified atmosphere, 37 °C, 5% CO2). The radiotracer was freshly produced on-site before the experiment. To assess nonspecific binding, the filter plates were incubated with the radiotracer alone. After incubation, the cells were washed by vacuum filtration with PBS (2 × 200 µL) through the plate. The filters were transferred into tubes using a commercial punch kit, and radioactivity was measured using a gamma counter (Wizard 2, PerkinElmer, Waltham, MA, USA). Radiotracer uptake was quantified as the percentage of added radioactivity per 100,000 cells. Nonspecific binding in all samples was < 0.1%.

### Synergy analysis

The synergy of drug action was calculated based on findings from the resazurin assay as described above. The degree of synergy was quantified using the Loewe Additivity model. This model assumes that the expected effect of a drug combination is as if a drug is combined with itself, considering the full dose-response relationships of the individual drugs. The four-parameter logistic regression (LL4) was used as the curve-fitting algorithm. This model provides a more accurate and nuanced interpretation of drug interactions, incorporating parameters such as EC50 and dose-response curves. A Loewe synergy score was derived to evaluate the combined effects, where deviations from the expected additivity indicate whether interactions are either synergistic or antagonistic.

### RNA isolation and qRT-PCR

Cells were trypsinized, pelleted and resuspended in 300 µl of Lysis Buffer with RNAsecure™ RNase Inactivation Reagent (Invitrogen, AM7005). RNA was isolated with RNeasy Mini Kit (Qiagen, 74104) according to the manufacturer’s protocol.

2 µg of RNA was reverse transcribed using High-Capacity cDNA Reverse Transcription Kit (Applied Biosystems, cat. no. 4368814) according to the manufacturer’s protocol. Then, 1 µL of 1:10 diluted cDNA for one reaction was used to perform qRT-PCR using TaqMan Fast Advanced Master Mix (Applied Biosystems, cat. no. 4444557) with TaqMan Gene Expression Assays for the respective genes: AR (Hs00907244_m1) and β-tubulin (Hs00742828_s1). Relative expression was calculated by the fold change method (ΔΔCt) with tubulin as endogenous control and normalized to the non-treated control.

### Protein isolation and Western blot analysis

Cells were harvested by trypsinization and pelleted by centrifugation at 500 x g for 5 min. Cell pellets were washed with ice-cold PBS and lysed in RIPA buffer (Sigma-Aldrich, St. Louis, MO, USA, #D8418) supplemented with protease (cOmplete™ Mini Proteasehemmer-Cocktail, Roche, #11836153001) and phosphatase inhibitors (PhosSTOP™, Roche, # 4906845001). The cell lysates were incubated on ice for 15 min. Subsequently, the lysates were centrifuged at 20,000 g for 20 min at 4 °C. The supernatants were collected, protein concentrations were determined by using the BCA (Thermo Scientific™, 23227) method, and samples were stored at −80° C. Equal amounts of each protein sample were separated by SDS-PAGE using 10% polyacrylamide gels. Proteins were then transferred onto nitrocellulose membranes (Cytiva, #10600001) using a Trans-Blot Turbo Transfer System (Bio-Rad, Hercules, CA, USA). The membranes were blocked with 5% BSA and incubated with primary antibodies (table below) overnight at 4° C. After 3 × 5 min washing, the membranes were incubated with HRP-conjugated secondary antibodies and visualized using an ECL detection system (Clarity™ Western ECL Substrate, #1705060) using the ChemiDoc XRS+ (Bio-Rad).

**Table:** 

Antibody	Dilution	Cat. number and company
TRβ	1:1000 in 5% BSA	Abx001332, Abbexa
AR	1:1000 in 5% BSA	ab74272, ab194196, Abcam
PSA	1:1000 in 5% BSA	5365 s, Cell Signaling
Nkx3.1	1:1000 in 5% BSA	#83,700, Cell Signaling
Tubulin	1:5000 in 5% BSA	#86,298, Cell Signaling
Actin	1:5000 in 5% BSA	#3700, Cell Signaling

### RNA-sequencing

RNA integrity was checked on the Fragment Analyzer using RNA Kit 15 nt (Agilent Technologies). 500 ng of total RNA was used as input for library preparation using QuantSeq 3′ mRNA-Seq FWD with UDI 12 nt Kit (v.2) (Lexogen) in combination with UMI Second Strand Synthesis Module for QuantSeq FWD. Quality control for library quantity and size distribution was done using QuantiFluor dsDNA System (Promega) and High Sensitivity NGS Fragment Analysis Kit (Agilent Technologies). Final library pool was sequenced on NextSeq 500 (Illumina) using High Output Kit v2.5 75 Cycles (Illumina) in single-end mode, resulting in an average of > 10 million reads per sample.

High-throughput RNA-Seq data were prepared using the Lexogen Quantseq forward Kit for Illumina with polyA selection and sequenced on Illumina sequencer (run length 1 × 75 nt). Bcl files were converted into Fastq format using bcl2fastq v. 2.20.0.422 Illumina software for base calling. Quality check of raw single-end fastq reads was carried out by FastQC [23]. The adapters and quality trimming of raw fastq reads was performed using Cutadapt v4.3 [24] with Illumina adapter trimming and parameters -m 35 -q 0,20. Trimmed RNA-Seq reads were mapped against the human genome (GRCh38) and Ensembl annotation release 111 using STAR v2.7.11 [25] as splice-aware short read aligner and default parameters except --outFilterMismatchNoverLmax 0.4 and --twopassMode Basic.

The differential gene expression analysis was calculated based on the gene counts produced using featureCounts from the Subread package v2.0 [26] and further analyzed by the Bioconductor package DESeq2 v1.34.0 [27]. Data generated by DESeq2 with independent filtering were selected for the differential gene expression analysis due to its conservative features and to avoid potential false positive results, and principal component analysis (PCA) was computed from gene expression normalized using DESeq2 Variance Stabilizing Transformation (VST). Genes were considered as differentially expressed based on a cut-off of adjusted *p*-value < 0.05 and log2(fold-change) ≥ log2(1.5) or ≤-log2(1.5). Clustered heatmaps were generated from selected top differentially regulated genes using R package pheatmap v1.0.12 [28], volcano plots were produced using ggplot2 v3.3.5 package [29] and MA plots were generated using ggpubr v0.4.0 package [30]. Venn diagrams were obtained by using the ggvenn package v0.1.10 [31].

### Cell cycle analysis

Cells were harvested by using trypsin and washed once with cold PBS (1X). The cells were then fixed with 70% ethanol and incubated on ice for 30 min. After centrifugation (800 g, 5 min), the cells were washed with PBS and resuspended in 100 µL of staining solution: propidium iodine (421301, BioLegend), RNAse 10 mg/mL, 0.01% Triton-X. After incubation at 37 °C for 40 min, samples were acquired with a BD FACSCanto II (Becton Dickinson, Franklin Lakes, NJ, USA) and analyzed with the FlowJo 7.6 software (BD Biosciences).

### Annexin V assay

Cells were harvested by using trypsin and washed twice with cold PBS (1X). Cells were then resuspended in Binding Buffer (1X) and stained with FITC Annexin V (R37174, Invitrogen™) and DAPI. Stained cells were incubated for 15 min at RT in the dark and analyzed by using FACSCanto II. Data analysis was performed using FlowJo 7.6 software (BD Biosciences). Cells treated with 5 µM bortezomib provided a positive control for apoptosis.

### Pharmacokinetics study

C57BL/6 mice were injected intraperitoneally (i.p.) with 3 mg/kg of NH-3. Blood samples were collected at the following time points: 10 min, 20 min, 30 min, 60 min, 240 min, and 480 min to EDTA-tubes (Mini-Collect K3EDTA tubes). Plasma was obtained by centrifuging the whole blood for 20 min at 1000 x g. For each sample, 8 µL of EDTA-plasma was precipitated using 232 µL methanol at −20 °C overnight. The precipitates were then centrifuged at 12,000 g for 10 min at 4 °C, and 180 µL of the supernatant was transferred to an HPLC vial. After drying under nitrogen flow, the samples were reconstituted in 120 µL of 33.3% mobile phase B (see below). Absolute quantification of NH-3 was determined using a matrix-matched external calibration curve.

Liquid chromatography-mass spectrometry (LC-MS) analysis was conducted using an Agilent Infinity II coupled to an Orbitrap Exploris 480 mass spectrometer operating in selected ion monitoring (SIM) mode. Chromatographic separation was achieved using water with 0.2% formic acid as mobile phase A and acetonitrile/methanol (90:10; v/v) with 0.2% formic acid as mobile phase B. The flow rate was set to 500 µL/min with an injection volume of 20 µL. The gradient elution began with an isocratic hold at 50% B for 0.2 min, followed by a linear increase to 100% B over 3 min. This was followed by a 1 min washout and 1.7 min equilibration phase. SIM was performed for NH-3 at 327.1602 m/z with a 1.6 m/z isolation window. The resolution was set to 90 000 full width at half maximum (FWHM) at 200 m/z, with a maximum injection time of 182 ms, an automatic gain control (AGC) target of 100%, an RF lens setting of 50%, and EASY-IC operating in scan-to-scan mode. Data was processed using Skyline, and pharmacokinetic parameters were calculated using R scripts.

### Mouse xenografts

Animal work was carried out according to ethical standards and was approved by the Medical University of Vienna and by the Austrian Federal Ministry of Science, Research, and Economy (GZ66.009/377-V/3b/2018). NSG mice were provided by the Institute of in vivo and in vitro Models (University of Veterinary Medicine, Vienna) and by the Core facility Laboratory Animal Breeding and Husbandry (Medical University of Vienna, Himberg). For 22Rv1 xenografts, NOD.Cg-Prkdcscid Il2rgtm1Wjl Tg(CMV-IL3,CSF2,KITLG)1Eav/MloySzJ mice were used, and for LNCaP xenografts, NOD SCID mice were used. To establish xenografts, NSG mice were injected subcutaneously with 22Rv1 (2 × 10^6^ cells per flank) and LNCaP (4 × 10^6^ cells per flank). Tumor dimensions were monitored biweekly by measuring with a caliper. Once tumors had reached a palpable size, mice were randomly assigned to treatment groups. Vehicle formulation was as follows: 5% (v/v) ethanol, 5% (v/v) Kolliphor EL, 30% (v/v) propylene glycol and 20% (w/v) HP-beta-CD in PBS (pH 7.4). Mice were treated daily with NH-3, enzalutamide, or both, diluted in vehicle and administered i.p. or by oral gavage (o.g.). Control mice received vehicle only. During the treatment period, mice were closely monitored for any signs of distress or adverse side effects. Tumor growth was monitored, and changes in tumor volume were recorded by measuring the length (L) and width (W) using a caliper. Tumor volume was calculated using the formula V = (L × W^2)/2. At the end of treatment, mice were euthanized, blood samples were taken by heart puncture, and xenograft tumors and organs were excised for further analysis. Statistical analysis was performed, and significance was determined at a *p* < 0.05.

### Histological analysis of tumor xenografts

Organs were fixed overnight in 4% phosphate-buffered formaldehyde solution (Roti^®^ Histofix, Carl Roth), dehydrated, paraffin-embedded, and cut into 2 μm consecutive organ or tumor sections. Sections were stained with Hematoxylin & Eosin G (according to a standard protocol). Slides were scanned using Vectra Polaris™ (Akoya Bioscences). Organ toxicity evaluation was performed by an animal pathologist. Tumor morphology was assessed by two independent expert pathologists.

### Blood parameters evaluation

Mouse blood collected by heart puncture was added to EDTA-tubes (Mini-Collect K3EDTA tubes). Plasma was obtained by centrifuging the whole blood for 20 min at 1000 x g. Blood parameters were assessed with an animal blood counter (Vet abc, scil animal care; Hitachi/Roche Cobas 4000 c311 Analyzer). Plasma concentrations of blood urea nitrogen (BUN), aspartate aminotransferase (AST) and alanine aminotransferase (ALT) were determined using a laboratory chemistry analyzer (IDEXX VetTest 8008, IDEXX Laboratories) and specific assays (04467388190, 04467493190, 04460715 190, cobas).

### Computational modeling

Protonation states of the amino acid side chains were estimated by using the H + + program [32]. The protein was solvated by water molecules, and a number of Na + and Cl- ions that represent the 0.154 M NaCl solution, and further sodium ions to neutralize the charge of the protein. To describe the intra- and intermolecular interactions within the periodic simulation box, the CHARMM36 force field was used for the biomolecules, the ligands, and the ions [33], whereas for water, the TIP3P model was employed [34]. Bonds to hydrogen atoms were constrained throughout the simulations. The ligand topologies were generated by using the CGenFF web server [35]. Molecular dynamics simulations were performed with Gromacs 2024.3.

To control the temperature and the pressure in the simulations, a modified Berendsen thermostat (reference temperature 310 K, time constant 0.1 ps) and a Berendsen barostat (reference pressure 1 bar, time constant 2 ps) were applied. The timestep was chosen to be 2 fs. Molecular docking calculations were performed at the Swissdock web server using Autodock Vina [36, 37].

### Statistical analysis

For datasets with a normal distribution, Student’s *t*-test was used for group comparison. If the distribution was not normal, Mann-Whitney *U* test was used. Statistical significance was defined as *p* < 0.05 unless stated otherwise. Significance levels are indicated as follows: *p < 0.05, **p < 0.01, ***p < 0.001, ****p < 0.0001.

## Results

### Thyroid hormone signaling drives PCa cell proliferation

**Fig. 1:**
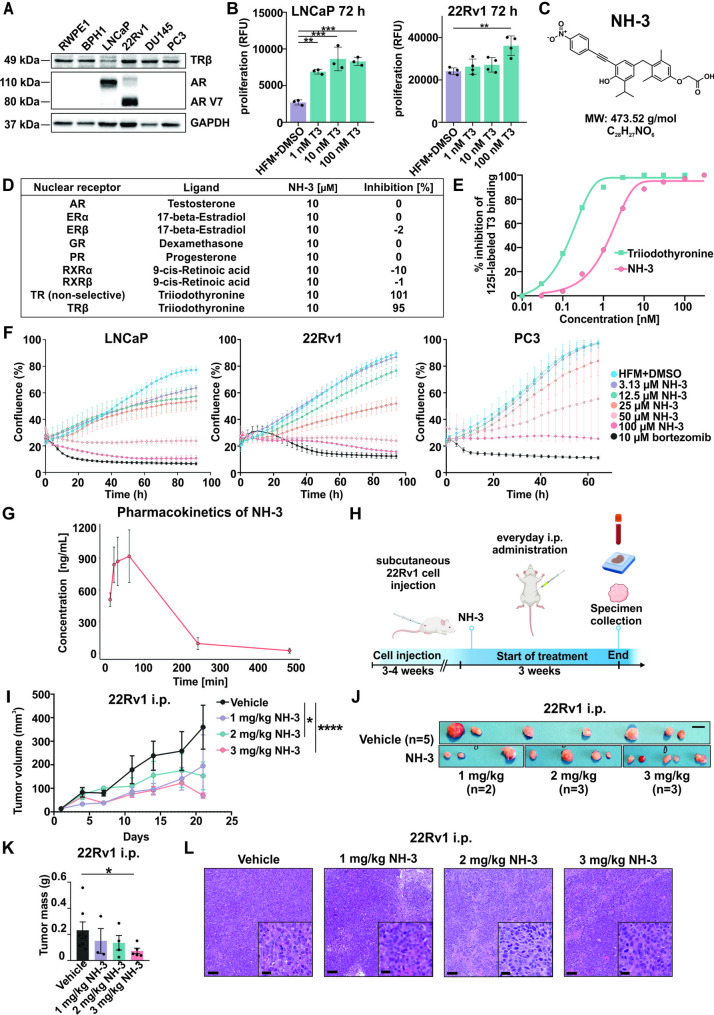
Treatment of PCa cells with TRβ-specific inhibitor NH-3 reduced PCa cell proliferation in vitro and xenograft tumor growth in vivo. (A) TRβ and AR protein expression in PCa cell lines RWPE1, BPH-1, LNCaP, 22Rv1, PC3, and DU145. GAPDH was used as a protein loading control, AR V7 – AR splice variant 7. (B) T3 stimulates LNCaP and 22Rv1 proliferation in hormone-free conditions in a dose-dependent manner. (C) Chemical structure of NH-3. (D) Percentage of inhibition seen in the nuclear receptor screen against AR, ERα, ERβ, GR, PR, RXRα, RXRβ, TR (non-selective), and TRβ and their respective ligands with 10 µM NH-3. (E) Inhibition of the binding of [I125]-radiolabeled T3 to TRβ by unlabeled T3 (green line) and NH-3 (orange line). Ki of T3 = 0.15 nM, Ki of NH-3 = 1.31 nM. (F) Reduced proliferation of LNCaP, 22Rv1, and PC3 cells as measured every 2 h upon post NH-3 treatment in hormone-free conditions, as calculated by the % cell confluence in each well. Bortezomib was used as a positive control by inducing cell death. (G) NH-3 serum concentration-time curve of 3 mg/kg i.p. treated C57BL/6 mice (n = 3). (H) Scheme of the NH-3 i.p. treatment in the 22Rv1 xenograft model. Created with BioRender.com. (I) 22Rv1 tumor volume during the treatment period. (J) Visual representation of the 22Rv1 xenografts treated i.p. with 1, 2, 3 mg/kg/day of NH-3. The black bar represents 1 cm. (K) Tumor masses after the treatment endpoint showing tumor growth inhibition in the treatment groups. (L) Representative H&E staining of 22Rv1 xenograft tumors showing no morphological differences. Bars (in the overview) are equivalent to 100 µm and bars (in the inserts) to 20 μm. Statistical analysis by 2-way ANOVA with multiple comparisons. Mean ± SD, *p < 0.05, **p < 0.01, and ***p < 0.001.

To investigate the role of TRβ in PCa, we analyzed TRβ protein expression within a panel of PCa cell lines. RWPE1 cells, derived from histologically normal prostate tissue, served as a non-malignant model, while BPH-1 cells, derived from benign prostatic hyperplasia (BPH), represented premalignant prostate tissue. LNCaP and 22Rv1 cells served as AR-positive models. Consistent with prior studies [38], only these two cell lines expressed the full-length AR, with the AR-V7 form present in 22Rv1 (Fig. 1A, middle panel), making them a model for CRPC. PC3 and DU-145 cells, which are AR-negative, were used as metastatic PCa cell lines. TRβ protein expression was detected in all the cell lines analyzed (Fig. 1A, upper panel). Given their AR expression profiles, LNCaP and 22Rv1 were selected for subsequent experiments, with 22Rv1 chosen specifically for its relevance as a CRPC model.

To determine whether TRβ signaling influenced proliferation, LNCaP and 22Rv1 cells were treated with T3 under hormone-free medium (HFM) conditions. In agreement with previous reports, T3 stimulation significantly increased the proliferation of LNCaP cells [11, 39, 40]. Notably, we extended these findings by demonstrating that 22Rv1 cells also showed increased proliferation at 72 h of T3 treatment (Fig. 1B). These results further support the role of thyroid hormone signaling in driving PCa cell proliferation, including for CRPC models.

### NH-3 is a selective ligand for TRβ

To investigate the role of TRβ in PCa cell proliferation, we used the synthetic TRβ-selective ligand NH-3 **(**Fig. 1C**)** [41]. NH-3, a potent and specific TRβ antagonist, was used in both cell culture and various animal models [42–44]. We first reexamined the specificity of NH-3 for TRβ antagonism to exclude possible additional interactions and off-target effects. Radioligand binding assays of TRβ, the TRβ binding partners retinoid X receptor alpha (RXRα), retinoid X receptor beta (RXRβ), and five hormone receptors - androgen receptor (AR), estrogen receptor alpha (ERα), estrogen receptor beta (ERβ), glucocorticoid receptor (GR), progesterone receptor (PR) *-* that are activated by cholesterol-derived hormones and have known roles in PCa [45] were chosen (Fig. 1D). In each assay, the binding of respective radioactively labeled ligand to its receptor was monitored in the presence of increasing NH-3 concentrations. NH-3 only led to inhibition of T3 binding to TRβ (IC50 = 1.68 nM), with no inhibition of any other ligand binding to their respective receptors (Fig. 1E).

### NH-3 reduced the proliferation of PCa cells in vitro

To assess in detail the impact of TRβ inhibition on PCa cell viability, IC_50_ values of NH-3 for the human PCa cell lines were measured (Suppl. Figure 1 A). Cells were treated with increasing concentrations of NH-3 in hormone-free (HFM) conditions, and cell growth was monitored every two hours using live cell microscopy. NH-3 treatment significantly reduced the cell layer coverage of AR-positive LNCaP and 22Rv1 cells and AR-negative PC3 at higher concentrations as compared to the vehicle control (Fig. 1F). Further analysis assessing the proliferation marker Ki-67 revealed a shift from Ki-67^high^ cells towards a Ki-67^low^ population, indicating suppressed cell proliferation rates (Suppl. Figure 1B). To confirm these findings, a resazurin assay was used as a cell viability readout based on metabolic activity. NH-3 treatment led to inhibition of LNCaP, 22Rv1, and PC3 cell viability in a concentration-dependent manner (Suppl. Figure 1 C, left panel). Importantly, to rule out the possibility that the observed growth inhibition resulted from TRβ ligand deprivation, NH-3 was also tested under full-media (FM) conditions (Suppl. Figure 1 C, right panel). Antiproliferative effects were observed for both cell lines, confirming NH-3 efficacy in physiological conditions.

These findings established TRβ blockade by NH-3 as a potent strategy to inhibit PCa cell proliferation, reinforcing the pivotal role of TRβ in modulating cellular growth processes in PCa.

### NH-3 treatment reduced 22Rv1 PCa cell xenograft growth

We assessed the in vivo pharmacokinetics of NH-3 in C57BL/6 mice with 3 mg/kg i.p. treatment and the subsequent LC-MS-based detection of NH-3 in mouse sera to establish the concentration-time curve (Fig. 1G). NH-3 showed a C_max_ value of 1008 +- 248 ng/mLthat was reached between 30 min and 60 min after drug injection, and the area under the concentration-time curve divided by dose (AUC/D) was 830 h*mg/mL. The biological half-life period was 1.06 +- 0.24 h, indicating efficient NH-3 uptake and fast clearance.

We extended our investigation of NH-3 function to PCa xenograft models. Given the strong effect observed for the 22Rv1 cell line and its relevance as a CRPC model, we first used this cell line for xenograft experiments. NSG mice were injected subcutaneously with 22Rv1 cells, and treatment was started once the tumors had reached approximately 100 cm³. For three weeks, mice received a daily i.p. injection of NH-3 at doses of 1, 2, or 3 mg/kg. Tumor size and body weight were monitored throughout the treatment period, and tumors, sera, and tissue samples were collected at study termination (Fig. 1H).

NH-3 treatment led to dose-dependent tumor growth inhibition (TGI), with 80% TGI at 3 mg/kg, 57% TGI at 2 mg/kg, and 46% TGI at 1 mg/kg. Notably, tumors in the 3 mg/kg group exhibited sustained growth suppression, with volumes remaining stable at approximately 125 mm³ until the study endpoint (Fig. 1I, J, K). Morphologically, all tumors showed high densities of cancer cells interspersed with rather thin stromal regions, but no further remarkable differences (Fig. 1L). NH-3 treatment was well tolerated, as indicated by the absence of any significant body weight changes (Suppl. Figure 1D). Serum analysis revealed no abnormalities in metabolic markers, ruling out potential liver or kidney toxicity (Suppl. Figure 1E). Histopathological examination of heart, lung, kidney, liver, spleen, and colon tissues showed no morphological abnormalities in the 3 mg/kg group compared to vehicle. These findings highlighted NH-3 as a potent and well-tolerated TRβ antagonist that effectively suppresses CRPC tumor growth in vivo.

**Fig. 2:**
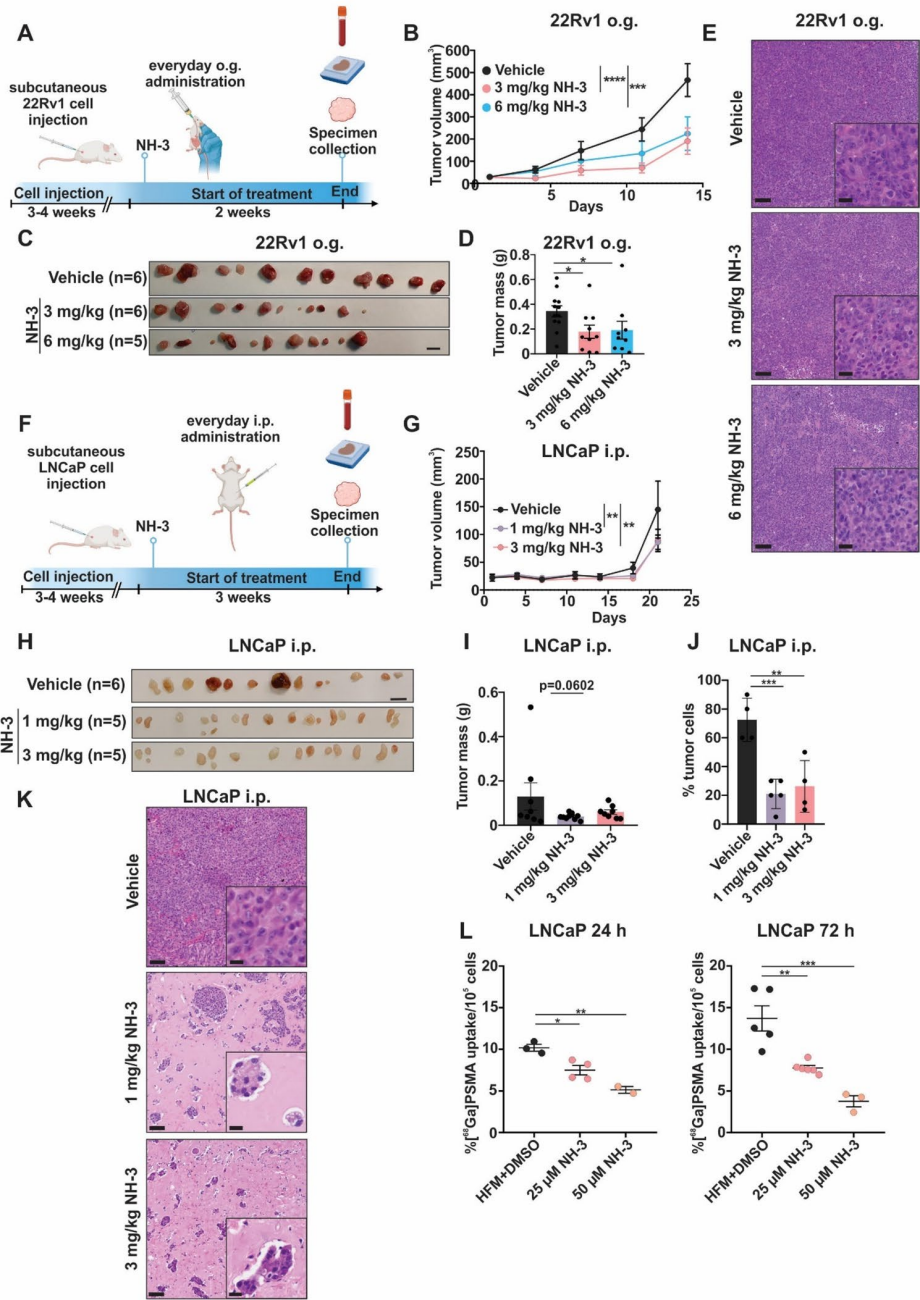
Inhibition of 22Rv1 and LNCaP PCa tumor growth by TRβ-specific inhibitor NH-3 *in vivo* and by different administration routes.** A** Scheme of NH-3 o.g. treatment in 22Rv1 xenograft model. Created with BioRender.com. **B** Reduced 22Rv1 tumor volumes during treatment. **C** Visual representation of 22Rv1 xenografts in mice treated with 3 and 6 mg NH-3/kg/day by o.g. The black bar is equivalent to 1 cm. **D** Reduced 22Rv1 tumor mass at the treatment endpoint. **E** Representative H&E staining of 22Rv1 tumors showing no obvious morphological differences. Bars (in the overview) are equivalent to 100 μm and bars (in the inserts) to 20 μm. **F** Scheme of NH-3 i.p. treatment in LNCaP xenograft mice model. Created with BioRender.com. **G** Reduced LNCaP tumor volumes during the treatment. **H** Visual representation of LNCaP xenografts treated with 1 and 3 mg NH-3/kg/day via i.p. administration. The black bar represents 1 cm. **I** Reduced LNCaP tumor masses at the treatment endpoint. **J** Percentage of tumor cells in LNCaP tumors treated with vehicle control, 1 and 3 mg/kg/day NH-3. **K** Representative H&E staining showing increased stromal areas in NH-3 treated LNCaP tumors. Bars (in the overview) are equivalent to 100 μm and bars (in the inserts) to 20 μm. **L **Gamma counter measurements of [^68^Ga]PSMA uptake in LNCaP cells treated with vehicle control, 25 µM, and 50 µM NH-3. Results are shown as % uptake and normalized to 10^5^ cells. Mean ± SD, *p < 0.05, **p < 0.01, and ***p < 0.001

NH-3 has been reported to be bioavailable for oral administration [41]. Therefore, we tested its antitumor efficacy when administered by o.g. After establishing 22Rv1 xenograft tumors, mice received daily NH-3 by o.g. for two weeks at 3 and 6 mg/kg, along with a vehicle control group (Fig. 2A). Tumor size and body weight were monitored as previously described. NH-3-treated mice exhibited 22Rv1 tumors with a significantly reduced mass and volume compared to controls (Fig. 2B, C, D). Both treatment concentrations resulted in 50–55% TGI, while 6 mg/kg did not show higher efficacy than 3 mg/kg, indicating a minimal effective dose beyond which no further enhancement of tumor reduction occurred. Again, tumors contained a high density of cancer cells and were morphologically similar across groups (Fig. 2E). Importantly, mice maintained stable body weight throughout the 2-week treatment period (Suppl. Figure 2 A). Serum analyses confirmed no signs of kidney or liver dysfunction (Suppl. Figure 2B). Histopathological evaluation revealed scattered lymphocytes in the portal areas of one animal each in the vehicle and 6 mg/kg, which are interpreted to be incidental findings. Kidney, spleen, lung, heart, and colon exhibited no lesions, regardless of group assignment (Suppl. Figure 2 C). Together, the above findings have shown that NH-3 inhibits PCa xenograft growth when administered either orally or i.p. and that continual dosing was well-tolerated with no detectable toxicity.

### NH-3 treatment reduced LNCaP PCa xenograft growth and PSMA uptake

The findings for the 22Rv1 xenograft model were validated using LNCaP xenografts. Following cell engraftment, mice received for three weeks daily i.p. injections of NH-3 at 1 and 3 mg/kg (Fig. 2F). At the treatment endpoint, tumors of the NH-3 treated groups were significantly smaller (Fig. 2G, H), and a trend towards reduction of tumor mass was observed (Fig. 2I). Treatments resulted in a 40% TGI for both tested NH-3 concentrations, indicating a slightly smaller effect compared to that seen for 22Rv1 xenografts. Histological evaluation based on H&E staining revealed highly hyalinized and desmoplastic stromal areas and strongly reduced tumor cell percentage for NH-3-treated mice compared to vehicle controls (Fig. 2J, K). NH-3 treated mice did not lose body weight throughout the experiment (Suppl. Figure 2D), serum analysis showed no signs of toxicity (Suppl. Figure 2E), and histopathological analysis did not reveal any signs of tissue degeneration or inflammation (Suppl. Figure 2 F).

Prostate-specific membrane antigen (PSMA) is a transmembrane glycoprotein that is highly expressed in PCa. It is widely used as a diagnostic marker for PCa, and its elevated expression is indicative of disease progression [38]. The LNCaP cell line expresses high levels of PSMA [46, 47] and therefore serves as a valuable model for PSMA research in PCa. Using a Gamma counter, we quantified the in vitro uptake of [^68^Ga]PSMA radiotracer in NH-3-treated LNCaP cells compared to non-treated cells. A dose-dependent reduction of radiotracer uptake was observed at 25 and 50 µM NH-3 concentrations after 24 h and 72 h treatment (Fig. 2L). This effect indicates an impact of NH-3 on PSMA-associated signaling pathways and tumor metabolism, which leads to reduced tumor growth. No unspecific radioligand binding was detected, confirming the specificity of [^68^Ga]PSMA under the experimental conditions. Together, these findings demonstrate that NH-3 inhibits PCa xenograft growth through both oral and i.p. administration, with well-tolerated dosing and no detectable toxicity. From the above data, we concluded that NH-3 reduced PSMA expression and activity.

### TRβ Inhibition affects the AR and AR-regulated gene expression

**Fig. 3:**
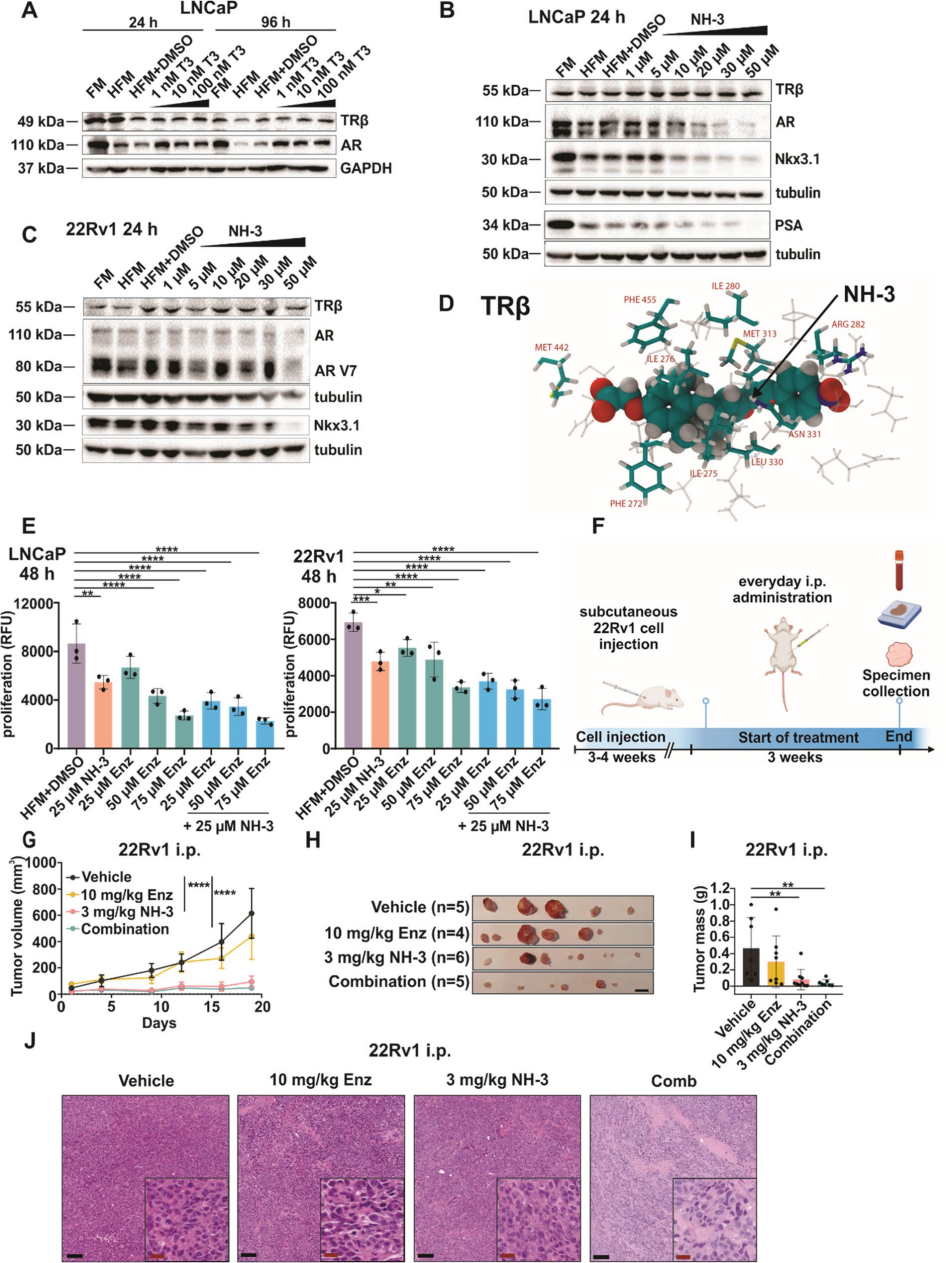
Selective TRβ blockade induces downregulation of AR and AR-regulated genes and is more effective than enzalutamide.** A** Western blot analysis of LNCaP cells treated with 1 nM, 10 nM, 100 nM T3 after 24 and 96 h. Representative protein expression of TRβ, AR, and GAPDH was used as a control; FM – full media, HFM – hormone-free media. **B** Western blot analysis of LNCaP cells treated with increasing NH-3 concentrations after 24 h. Representative blot showing expression of TRβ, AR, PSA, Nkx3.1; GAPDH and tubulin as loading controls. **C** Western blot analysis of 22Rv1 cells treated with increasing NH-3 concentrations after 24. Representative protein expression of TRβ, AR, PSA, Nkx3.1, GAPDH, and tubulin as loading controls. **D** Binding of NH-3 to TRβ (2PIN structure), as obtained from molecular docking calculations and subsequent molecular dynamics simulations. The residues of TRβ that are adjacent to NH-3 are highlighted and labelled. **E** Proliferation (RFU) of LNCaP and 22Rv1 cells treated with 25 µM NH-3, 75, 57, 25 µM enzalutamide and combination treatment; Enz – enzalutamide. **F** Scheme of NH-3, enzalutamide and combination i.p. treatment in the 22Rv1 xenograft model. Created with BioRender.com. **G** Reduced tumor volumes during the drug treatments. Combined NH-3 and enzalutamide treatment leads to complete inhibition of tumor growth. **H** Visual representation of 22Rv1 xenografts treated i.p. with 3 mg/kg/day NH-3, 10 mg/kg/day enzalutamide, and a combination of both at the treatment endpoint. The black bar represents 1 cm. **I** Reduced tumor mass after the treatment. **J** Representative H&E staining of 22Rv1 tumors showing high tumor cell density and slight enhancement of stromal regions with combinatorial treatment. Bars (in the overview) are equivalent to 100 μm and bars (in the inserts) to 20 μm. Mean ± SD, *p < 0.05, **p < 0.01, and ***p < 0.001

AR-signaling is the pivotal pathway that drives PCa progression and the transition to CRPC disease [38]. T3 was observed to stabilize AR levels in LNCaP cells [39]. We examined the influence of T3 stimulation on TRβ and AR expression in LNCaP cells under HFM conditions. While TRβ protein levels remained stable, HFM led to reduced AR expression, which was fully restored upon T3 treatment at later time points (Fig. 3A).

We evaluated whether NH-3 treatment modulates TRβ, AR, and its downstream target gene expression. TRβ protein levels remained stable in LNCaP cells with increasing concentrations of NH-3 (Fig. 3B, C). These findings confirmed that NH-3 operates via inhibition of TRβ activity rather than by a ligand-induced degradation mechanism, as observed for other nuclear receptor modulators [48–50]. Notably, NH-3 treatment led to reduced AR protein levels after 24 h (Fig. 3B, C), which was sustained at 72 h (Suppl. Figure 3 A, B). AR-V7 downregulation was also observed for 22Rv1 cells. Additionally, the AR downstream targets Nkx3.1 and PSA (in LNCaP) were downregulated in a dose-dependent manner (Fig. 3B, C). Analysis of AR transcript levels revealed no significant changes in LNCaP cells after NH-3 treatment (Suppl. Figure 3 C). These findings showed that TRβ antagonism by NH-3 suppresses AR signaling. Even so, this pattern does not conform to canonical transcriptional gene regulation, in which a decrease in mRNA expression leads to a corresponding reduction in protein levels.

To assess how NH-3 binds to TRβ, we performed molecular docking calculations, supplemented with a sequence of molecular dynamics (MD) simulations. Two TRβ protein structures obtained by X-ray diffraction were chosen from literature, 3GWS [51] and 2PIN [52]. Each of these structures contain a ligand, the former triiodothyronine (T3), the latter the inhibitor metabolite tiratricol (4HY), clearly defining the native ligand binding site of TRβ. After an initial 10 ns MD run to remove all energetic hotspots from the system, T3 and 4HY were cut out of the proteins, and the NH-3 molecule was docked into the binding site. Reassuringly, the docking resulted in an identical orientation of the drug inside the protein in both cases. The structure is shown in Fig. 3D, with the amino acid side chains adjacent to NH-3 highlighted and labelled. The binding energy was found to be significant, ΔE = −8.7 and − 7.0 kcal mol-1 for 3GWS and 2PIN, respectively. Performing 20 ns of MD simulations on the system allowed further adjustment of the conformations of the biomolecules and NH-3 to ensure an even stronger binding. The strength of the interaction can be represented by the development of interaction energies between the proteins and the drug, averaging at ΔE_int_ = −73.5 and − 69.3 kcal mol-1 through the last 10 ns of the simulations for 3GWS and 2PIN, respectively. Cutting out NH-3 from the resulting structures and performing the docking calculations anew resulted in even more pronounced binding energies, amounting to ΔE = −11.3 and − 11.5 kcal mol-1. The similarity in interaction and binding energies obtained from two different starting structures supports the experimental finding of a strong interaction between TRβ and NH-3.

To confirm the observed effects of NH-3 on AR expression, we made use of another TRβ antagonist, 1-850 (Suppl. Figure 3 C). 1-850 also inhibited the proliferation of LNCaP and 22Rv1 cells (for IC50 curves see in Suppl. Figure 3D). Treatment of LNCaP cells with 1-850 similarly reduced AR and PSA expression (Fig. 3E), emphasizing the role of TRβ in maintaining AR signaling.

We investigated the therapeutic potential of combining NH-3 with the AR antagonist enzalutamide in LNCaP and 22Rv1 cells. Enzalutamide alone reduced the proliferation of both cell lines in a dose-dependent manner, and co-treatment with NH-3 enhanced this effect (Fig. 3E). Synergy analysis experiments demonstrated an additive growth inhibition across dose-response matrices for both cell lines, as revealed by distinct peaks in 3D synergy maps (Suppl. Figure 3 F). Given the growth-blocking synergy observed in vitro, we evaluated NH-3 and enzalutamide in vivo using NSG mice engrafted with 22Rv1 cells (Fig. 3F). The most effective dose of NH-3 was chosen for combinatorial treatments. Mice treated with NH-3 alone and with a combination of NH-3 and enzalutamide exhibited significantly smaller tumor volumes and masses (Fig. 3G, H, I) compared to control groups. Combination therapy resulted in enhanced tumor growth suppression relative to single-agent treatment. Morphologically, the tumors contained high numbers of cancer cells, interspersed with stromal septa, which were slightly enlarged for the combinatorial treatment group (Fig. 3J). Importantly, body weights remained stable throughout the 3-week treatment (Suppl. Figure 3G), and serum analyses revealed no kidney or liver toxicity (Suppl. Figure 3 H). Histological evaluation revealed moderate vacuolation of the proximal tubular epithelium in the kidneys of all animals treated with a combination of NH-3 and enzalutamide. In the other groups, the tubular epithelium was normal or showed only mild vacuolation. No lesions were detected in the liver, spleen, lung, heart, and colon, regardless of group assignment. The vacuolation of the tubular epithelium appears to be associated with the combined treatment. Single treatments with either NH-3 or enzalutamide did not result in these lesions (Suppl. Figure 3b. J). The enhanced antitumor effects seen for the combination of NH-3 with enzalutamide support the potential use of NH-3 in a combination therapy that would target both, the TRβ and AR pathways.

### TRβ Inhibition by NH-3 leads to dysregulation of genes involved in PCa cell growth

**Fig. 4:**
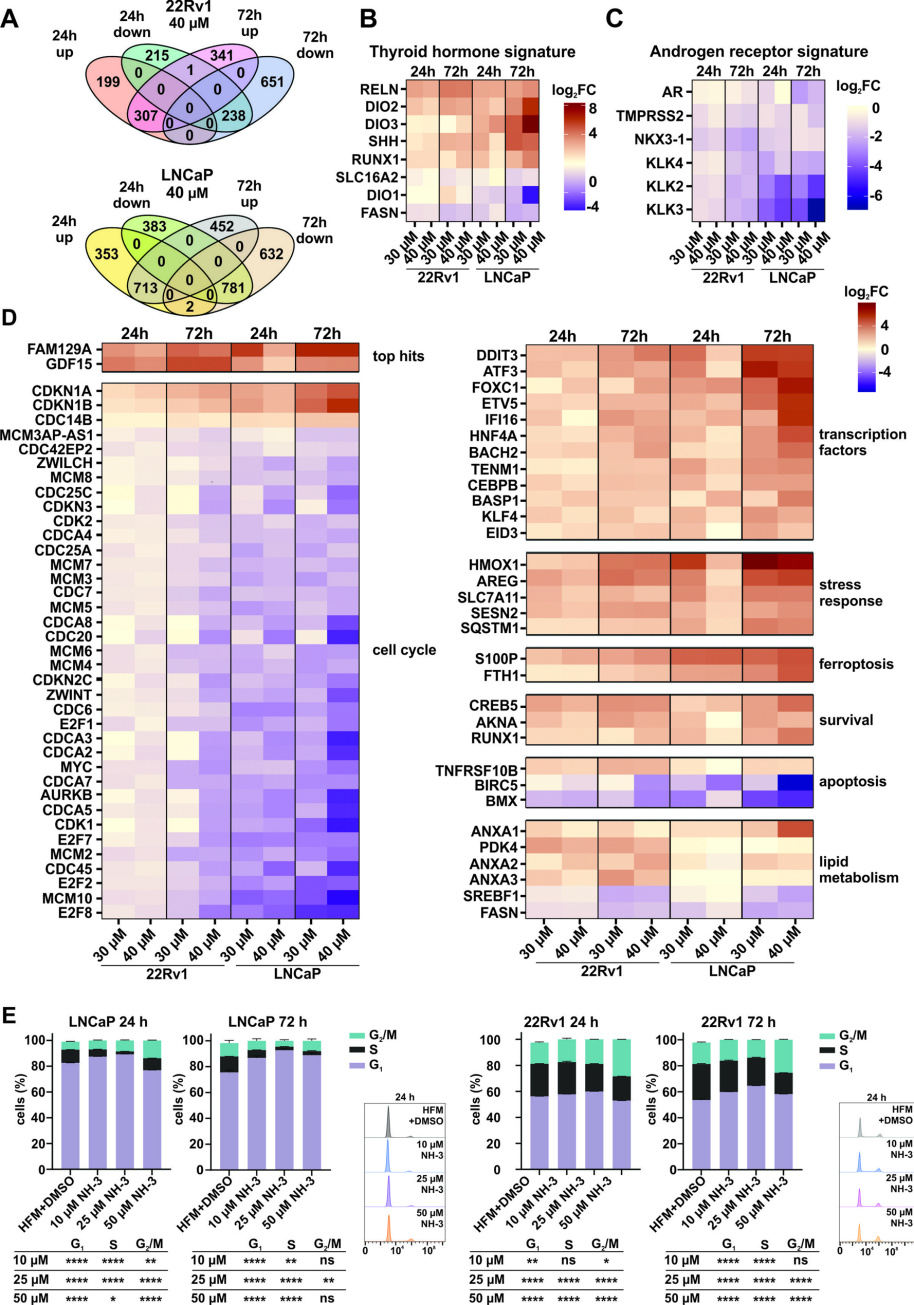
Genes and pathways affected in 22Rv1 and LNCaP cells upon TRβ inhibition by NH-3.** A** Venn diagrams showing the overlap between up- and downregulated genes in 22Rv1 and LNCaP cells treated with 40 µM NH-3 for 24 and 72 h. Genes were ranked based on adjusted *p*-values (FDR < 0.05) and log2 fold-change thresholds (|log2FC| ≥ 1.5), and subsequently grouped according to their associated biological pathways using KEGG pathway enrichment tools. **B** Heatmap representing dysregulated TRβ-related gene signature and (**C**) AR gene signature in 22Rv1 and LNCaP cells treated with NH-3 at two concentrations and determined after two timepoints. Shown are log2fold change gene expression values compared to the hormone-free medium control. **D** The most deregulated genes in LNCaP and 22Rv1 cells treated with NH-3 were sorted by pathways. **E** Cell cycle analysis of LNCaP and 22Rv1 cells treated with NH-3. Mean ± SD, *p < 0.05, **p < 0.01, and ***p < 0.001

To gain further insight into TRβ-driven processes in PCa, we performed a transcriptome profiling of LNCaP and 22Rv1 cells treated with NH-3 at two concentrations and time points. A cluster analysis determined grouped transcriptomes of LNCaP and 22Rv1 cells, with clear separation between control and NH-3 treated samples (Suppl. Figure 4 A). NH-3 treatment induced broad transcriptional changes, with up to two thousand genes significantly up- or downregulated in both cell lines, with a slightly stronger effect on fold-deregulation in LNCaP cells (Suppl. Figure 4B). Overall, the most deregulated transcripts overlapped substantially between the two cell lines, suggesting a shared mode of TRβ activity. Longer duration and higher concentration of NH-3 treatment led to stronger transcriptomic deregulation in both cell lines, as visualized in the Venn diagrams (Fig. 4A). Gene set enrichment analysis (GSEA) of the differentially expressed genes (DEGs) identified common but also cell line-specific mechanisms (Suppl. Figure 4 C).

TRβ and AR-associated gene signatures were strongly deregulated (Fig. 4B). While *THRB* mRNA levels remained unchanged, disturbance of TH synthesis and degradation pathways was observed, as indicated by downregulation of *DIO1*, and upregulation of *DIO2* and *DIO3* (Fig. 4B). Deregulation of additional TH transport and signaling genes (*SLC16A2*, *RELN*, *SHH*, *RUNX1*, *FASN*) further confirmed effective TRβ blockade by NH-3 (Fig. 4B). Importantly, *AR* and AR-regulated transcripts (*NKX3.1*, *TMPRSS2*, *KLK3*, *KLK2*) were downregulated (Fig. 4C), implicating a crosstalk with AR signaling. A pronounced stress response was observed, marked by upregulation of *ATF3*, *AREG*, *GDF15* and *FAM129A*. Genes mediating cell cycle blockade (*CDKN1A*, *CDKN2B*, *E2F1*) were upregulated, whereas drivers of cell progression (*CDK1*, *MCM10*, *MYC*, *RRM2*, *TYMS*) were downregulated (Fig. 4D). Moreover, lipid metabolism pathway genes were suppressed in both cell lines (Fig. 4D). To validate the transcriptomic findings, we conducted cell cycle and apoptosis assays under NH-3 treatment conditions. Consistent with the RNA-seq data, NH-3 induced cell cycle arrest in LNCaP and 22Rv1 cells (Fig. 4E) and enhanced apoptosis in LNCaP cells (Suppl. Figure 4D). Taken together, these results demonstrate that TRβ blockade disrupts multiple oncogenic pathways, linking TRβ activity to AR signaling, stress adaptation, cell cycle control, and metabolic regulation in PCa cells.

### Thyroid hormone signaling pathway genes have frequent mutations in PCa patients

**Fig. 5:**
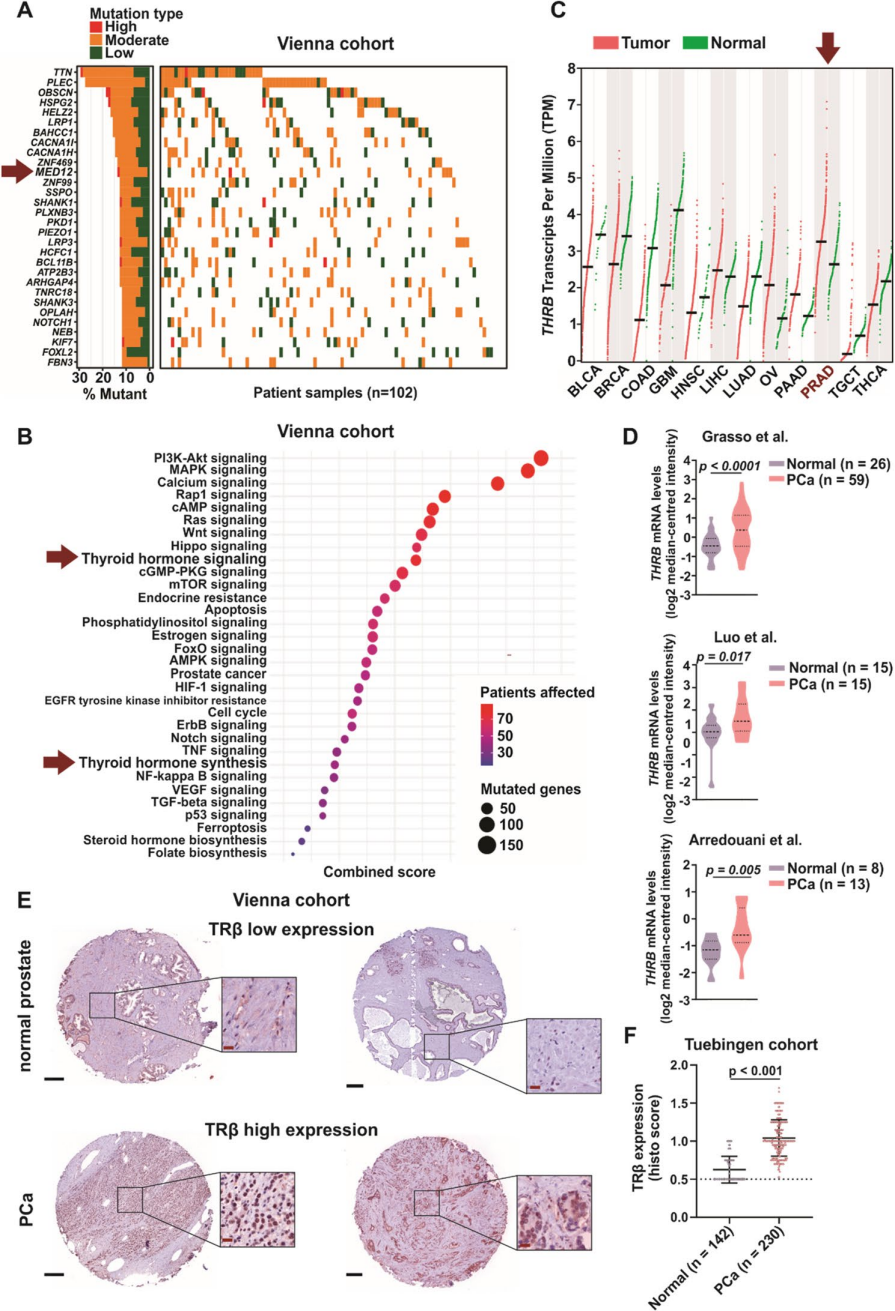
Enrichment of SNVs in thyroid hormone signaling pathway genes and enhanced thyroid hormone receptor beta mRNA (*THRB*) and TRβ protein expression in PCa patient cohorts. **A** Waterfall plot of the most frequently mutated genes derived from whole-exome sequencing of 102 treatment-naïve PCa patients who had undergone radical prostatectomy (Vienna cohort). **B** Bubble plot of signaling pathways in which the SNV-affected genes from the same PCa patient cohort are enriched. Each bubble represents a pathway; the size corresponds to the number of genes affected by SNVs within that pathway, and the color indicates the number of patients affected. The x-axis represents the combined score, representing the significance and impact of the pathway mutations. Mutation impact ratings (high, moderate, low) were determined according to the Ensembl Variant Effect Predictor (VEP) classification. **C** *THRB* transcript levels across different cancer types (BLCA – Bladder urothelial carcinoma, BRCA – Breast invasive carcinoma, COAD – Colon adenocarcinoma, GBM – Glioblastoma multiforme, HNSC – Head and neck squamous cell carcinoma, LIHC – Liver hepatocellular carcinoma, LUAD – Lung squamous cell carcinoma, OV – Ovarian serous cystadenocarcinoma, PAAD – Pancreatic adenocarcinoma, PRAD – Prostate adenocarcinoma, TGCT – Testicular germ cell tumors, THCA – Thyroid carcinoma), source: GEPIA. **D** *THRB* mRNA levels extracted using the Oncomine database from three different datasets: Grasso et al. (Normal *n* = 26, PCa *n* = 59), Luo et al. (Normal *n* = 15, PCa *n* = 15), Arredouani et al. (Normal *n* = 8, PCa *n* = 13). **E** Representative IHC staining of TRβ in normal prostate and PCa tissue, low expression depicts H-score 20, high expression depicts H-score 300. Black bars are equivalent to 250 μm and red bards to 20 μm. **F** Histological scoring of IHC staining of TRβ showed significantly higher TRβ expression in PCa patients (*n* = 230 slides) compared to healthy control tissue (*n* = 142 slides) (Tuebingen cohort)

We examined whether the significance of the TH signaling pathway is reflected by mutations in PCa. Genetic alterations in prostate tissue samples from a cohort of treatment-naïve PCa patients who underwent radical prostatectomy (*n* = 102) were analyzed by whole-exome sequencing (WES) [18]. The analysis involved the detection of single-nucleotide variants (SNVs) and the assessment of their impact on the associated pathways. Mutations were categorized into low, moderate, and high impact (Ensembl Variant Effect Predictor). No genetic changes were detected in the *THRB* gene. However, MED12, a key component of the thyroid hormone receptor-associated protein complex, was among the ten most frequently mutated genes (Fig. 5A), aligning with literature findings regarding its role in PCa [53, 54]. Furthermore, a gene set enrichment analysis using KEGG database annotation revealed that “thyroid hormone signaling” ranked ninth among cancer-related and hormonal signaling pathways most affected by mutations (Fig. 5B). Notably, mutations were identified in 79 genes within this pathway, with 74.1% of patients harboring single nucleotide variants (SNVs) in these genes (76 out of 102 cases). In addition, “thyroid hormone synthesis” ranked among the most affected pathways, further indicating the importance of TH signaling in PCa pathophysiology [55].

Mutations within the TH signaling and TH synthesis genes were found to be distributed across different tumors rather than accumulating in individual patients, suggesting that TH activity has a broad impact in PCa (Suppl. Table 1). A comparative search of the SNVs in the Cosmic database revealed variants previously described, as well as a multitude of SNVs that have not been described (Suppl. Table 1). Many of those have the potential to affect protein function: In the TH signaling genes, *MED12*, a subunit of the Mediator complex that regulates TH-specific transcription, harbored 11 non-silent SNVs, two of which are yet undescribed. One leads to R585X, creating a potential arginine modification site. The second, K1086M, produces a lysine, which is a potential sumoylation site. Both alterations can affect protein function and interactions. Among the known mutations, R1636C is located in the interaction domain with CTNNB1 and GLI3, while R1899Q affects an arginine modification site. These mutations suggest that MED12 cofactor interaction may be significantly altered in PCa. *MED16* harbored five non-silent variants, with one already described. Three of the four novel alterations are located in WD regions (R52H: WD1. S150L: WD3. G223S: WD5), which form the N-terminal protein interaction domain, suggesting potential alterations in MED16 function. A range of TRβ-associated transcriptional coactivators and corepressors were affected: Coactivator *CREBBP* contained four non-silent alterations, three of which are novel. These variants accumulated in the C-terminal region, which is involved in transcriptional regulation. The novel variant R1828C is located in the zinc finger region, and could lead to DNA-binding alterations. *NCOR1*, the main corepressor of TRβ, harbored two novel alterations, one of which located in the coiled coil region, that plays a crucial role in protein-protein interaction. Corepressor *NCOR2* harbored three novel alterations, all of which located at C-terminal end, that contain the nuclear receptor interacting domains, particularly binding to TRs and RARs. Nuclear receptor coactivator 3, *NCOA3*, harbored one novel alteration, generating a deletion. The main interaction partner of TRβ, *RXRB*, harbored one novel alteration in the ligand binding domain (LBD). Histone acetyltransferases that closely cooperate with TRβ were found affected: *EP300* (p300, *KAT3B*) harbored three non-silent alterations, two of which are novel, one insertion (Q2110fs) is located in the NCOA2 interaction domain, which may lead to altered TRβ-mediated gene transcription. *KAT2A* harbored two novel mutations in the acetyltransferase catalytic domain. Regarding the genes involved in TH synthesis (Suppl. Table 1), a significant number of SNVs were observed in *GNAS*. GNAS plays a crucial role in TH synthesis by forming the alpha subunit of the stimulatory G protein (Gsα), which is a key component in the TSH receptor signaling pathway, which is essential for regulating TH production. *GNAS* harbored six non-silent variants, one known and five novel, all in regions with undefined functions. LRP2, the main thyroglobulin uptake protein, harbored five non-silent SNVs, all of which were located in the extracellular domain. Furthermore, known variants were detected in *DUOX1* (one each in the extracellular, transmembrane and cytoplasmic regions), *IYD* (one in a non-defined region), and *TG* (one in a TG domain), which are enzymes involved in TH synthesis. The TH transporters *SLCO1C1*, *SLC16A10* and *SLC16A2* each contained one known SNV, all of which were located in the transmembrane domains. Overall, these mutations may affect modulation of TRβ-dependent signaling and impact TH availability in PCa cells.

We next analyzed *THRB* mRNA levels in publicly available human PCa transcriptome datasets. In Gene Expression Profiling Interactive Analysis (GEPIA), *THRB* transcript levels in tumors were higher compared to normal counterparts (Fig. 5C). From the Oncomine database, upregulation of *THRB* mRNA was observed across three distinct PCa cohorts compared to normal prostate tissues (Fig. 5D). We also examined *THRB* mRNA expression data from primary PCa (*n* = 739) and normal prostate tissues (*n* = 174) in the Prostate Cancer Transcriptome Atlas. We again observed that *THRB* transcript levels were upregulated when comparing primary tumors to controls (Suppl. Figure 5 ).

TRβ protein levels were assessed in an independent cohort of PCa biopsies using immunohistochemical (IHC) staining and scoring (Gleason scores 3–7) to complement the transcriptomic data. Significantly higher TRβ protein expression was observed in malignant tissues compared to adjacent normal tissues (Fig. 5F), corroborating the transcriptomic findings and suggesting a tumor-promoting role for TRβ [55]. Taken together, the above findings reveal widespread alterations in the thyroid hormone signaling pathway at the mRNA and protein levels in PCa. Elevated TRβ expression correlates with tumor occurrence, reinforcing its potential role as a biomarker and therapeutic target in PCa (Fig. 5).

## Discussion

The identification of the TRβ as a critical regulator of PCa progression marks a major advance in our understanding of hormonal modulation in cancer biology. Despite its recognized importance, the effects of using specific agonists or antagonists to target TRβ in PCa have not been thoroughly investigated. Here, we have tested the hypothesis that TRβ plays a central role in PCa cell growth and progression. We have shown that specific blockade of TRβ inhibits AR-driven and other signaling pathways that are associated with tumor proliferation. The activity of TRβ plays a role in AR expression, thereby affecting its downstream targets and contributing to the regulation of cellular growth and survival. These findings position TRβ as a novel and promising therapeutic target in PCa.

TRβ has emerged as a versatile and clinically relevant pharmacological target, offering promising therapeutic opportunities through its activation and inhibition. It has dual functionality, either activating or repressing gene expression, which allows precise modulation of signaling pathways involved in diverse pathophysiological conditions, including metabolic diseases and cancer. The selective TRβ agonist resmetirom highlights the therapeutic potential of TRβ activation in hepatocytes. It was effective in treating non-alcoholic steatohepatitis (NASH) in a Phase 3 trial (MAESTRO-NASH) [14]. Resmetirom achieved NASH resolution and fibrosis improvement [56], leading to FDA approval in March 2024 [15]. These reports underscore the potential of TRβ activation for metabolic regulation and open avenues for the repurposing of TRβ agonists in diseases in which stimulation of TRβ leads to beneficial changes in gene expression. In contrast to TRβ activation, its inhibition offers a novel strategy for targeting cancers in which TRβ activity contributes to tumor aggressiveness, as in this study, through crosstalk with AR-driven PCa growth. This crosstalk and other mechanisms induced by TRβ remain to be elucidated. The selective TRβ antagonist NH-3 competitively binds to the ligand binding domain (LBD) with high affinity and effectively antagonizes T3 activity in luciferase assays [42, 57]. Demonstrating specificity and physiological effects characteristic of TRβ blockade, NH-3 has shown broad applicability across human cell lines and animal models [43, 44, 58, 59]. In this study, NH-3 was rigorously tested for specificity to rule out interference with other nuclear receptors, including AR-related pathways that are critical in PCa [60]. Molecular docking confirmed its selective binding to TRβ. While these findings support the specificity of NH-3, potential off-target effects cannot be completely excluded and warrant further investigation.

We observed that the action of NH-3 does not lead to TRβ downregulation at the protein level in PCa cells, thus preserving receptor integrity. This aligns with previous reports that NH-3 disrupts TRβ activation by promoting the release of the corepressors NCOR1 and NCOR2 (SMRT) without recruiting coactivators such as NCOA1 (SRC-1), NCOA2 (GRIP-1) or MED1 (TRAP220) [41, 42, 61]. This mechanism supports a model in which NH-3 disrupts TRβ coactivator interactions and inhibits downstream signaling pathways. We showed that NH-3 exhibit potent anti-proliferative activity in both, hormone-depleted and hormone-supplemented conditions, demonstrating that its effects are independent of ligand depletion. The delayed onset of action suggests involvement in the regulation of protein synthesis and broader transcriptional control. In PCa models, NH-3 significantly reduced cell proliferation, supporting its role as a promising therapeutic agent for AR-driven and CRPC.

AR has a short half-life and requires prolonged receptor occupancy for stability [62]. T3 stabilizes AR independently of androgens, and consequently, we observed AR degradation under hormone-free conditions. In addition, T3 enhances AR signaling and stimulates PSA expression and secretion [16] and affects AR expression in several models, including Sertoli cells in hypothyroid rats [63] and Harderian gland cells in male hamsters [64]. In LNCaP cells, T3 increased *KLK3* mRNA levels [12, 39, 65] while knockdown of CRYM, a T3 scavenger protein, reduced PSA secretion [16]. PSA, a marker of PCa progression, correlates with sustained AR activity, and its suppression reflects the efficacy of androgen deprivation therapy (ADT). Elevated serum T3 levels in PCa patients with high PSA [66] and decreased TH levels in ADT-treated patients [67] suggest a systemic interplay between TH and AR signaling. In silico promoter studies further suggest a combined or mutual regulation of AR- and TR-regulated genes by DHT and T3 [68], highlighting the role of T3 in supporting AR-driven processes and emphasizing its relevance in PCa progression. Consistent with this, we showed that NH-3 blockade of TRβ downregulated AR and its targets, Nkx3.1 and PSA, in both, androgen-dependent LNCaP and independent PTEN-positive 22Rv1 cells. Synergy maps revealed a strong sensitivity of LNCaP cells to low doses of enzalutamide, with peak effects observed at mid-range concentrations when combined with NH-3. For 22Rv1 cells, enzalutamide acted as an enhancer, amplifying the ability of NH-3 to downregulate AR signaling. *In vitro* experiments confirmed that NH-3 effectively inhibits AR activity and reduces Nkx3.1 and PSA expression in PCa cells.

NH-3 treatment led to a significant reduction of PCa tumor growth in NSG xenograft mouse models, consistent with other studies that have shown that modulation of various NRs has profound effects on tumor dynamics in vivo [40, 69, 70]. Treatment with NH-3 for three weeks did not cause recognizable side effects, although long-term studies are required to assess potential adverse effects.

Analysis of the effect of TRβ blockade at the global gene expression level showed a strong overlap of deregulated transcripts in both LNCaP and 22Rv1 cell lines, albeit with lower fold-changes and to a lesser extent in 22Rv1 cells. This suggests that LNCaP cells may depend more on TRβ function. Whilst there were several cell-intrinsic differences, the most strongly deregulated transcripts and pathways were essentially the same.

Treatment with NH-3 did not lead to changes in TRβ expression in PCa cells, at either the mRNA or protein level, indicating the absence of a disruption of its protein function. Important TRβ downstream genes were deregulated, which further confirms that TRβ activity was specifically blocked by NH-3. The significant impact of NH-3 on PCa cell growth highlights the importance of genomic TRβ activity.

Whole-exome sequencing (WES) revealed that early-stage PCa harbors mutations in previously described genes such as *SPOP* and *MED12* [53], validating that our cohort is representative. In contrast to previous reports suggesting a tumor-suppressive role of *THRB* due to deletions and mutations in various cancers [71–73] we did not detect significant SNVs in *THRB*, suggesting an intact TRβ structure. Nevertheless, 75% of treatment-naïve PCa samples showed perturbations in TH signaling and synthesis pathways, suggesting that an early selective advantage is conferred by TH activity. To date, alterations in thyroid hormone signaling have rarely been described in PCa, as most transcriptomic and proteomic analyses assess expression rather than function.

Our analyses revealed that *THRB* mRNA and TRβ protein levels were upregulated in tumor samples compared to normal controls in several cohorts. Altered TRβ expression has also been observed in other cancers, including colorectal cancer [74], breast cancer [75, 76] and head and neck squamous cell carcinoma [77]. These studies also reported an adverse survival correlation with nuclear or cytosolic TRβ localization, underscoring the need to distinguish between nuclear and cytosolic TRβ expression and highlighting the role of intracellular T3 availability in shaping the regulatory effect of TRβ on tumor progression.

## Conclusions

In this study, we demonstrate for the first time that activated TRβ significantly affects AR activity and promotes PCa cell growth. Specifically, we show that TRβ activation drives PCa cell growth and regulates AR-dependent processes. Importantly, we highlight the therapeutic potential of targeting TRβ with the compound NH-3, which shows high efficacy against PCa cells. Our findings underscore the broader significance of TRβ activation as a fundamental oncogenic mechanism, particularly in the context of CRPC. The synergistic inhibition of cell proliferation observed with NH-3 and enzalutamide for both LNCaP and 22Rv1 cells suggests a promising combinatorial approach to enhance AR inhibition. Notably, NH-3 demonstrated potent activity under ADT conditions, where impaired AR signaling amplifies the dependence on TRβ-mediated pathways, positioning TRβ as an essential factor for sustained PCa progression.

We propose from the findings of this study that TRβ-selective antagonists, such as NH-3, alone or in combination with enzalutamide, represent a novel and effective therapeutic strategy for PCa, particularly CRPC. The dual targeting of AR through distinct mechanisms may reduce the likelihood of resistance development, a pervasive challenge in current treatment paradigms. Nevertheless, future studies are needed to validate these findings and elucidate the full spectrum of TRβ oncogenic roles and its potential as a therapeutic target. This work fundamentally advances our understanding of PCa biology by highlighting TRβ as a critical factor in cancer progression, offering new avenues for combination therapies to address unmet clinical needs in advanced PCa.

## Supplementary Information

Supplementary Material 1.

Supplementary Material 2.

## Data Availability

The datasets supporting the conclusions of this article are included within the article and its additional Supplementary Materials. Further, the following publicly available datasets were used: The Cancer Genome Atlas (TCGA) (https://www.cancer.gov/ccg/research/genome-sequencing/tcga), the Oncomine Research Premium Edition database (Thermo Fisher, Ann Arbor, MI), and the prostate cancer transcriptome published by Theurillat et al. (22).

## Abbreviations

AUC/D: Area under the curve divided by dose
AR: Androgen receptor
CRPC: Castration–resistant prostate cancer
CRYM: µ–crystallin
ER: Estrogen receptor
FM: Full media
GR: Glucocorticoid receptor
HFM: Hormone–free media
IHC: Immunohistochemistry
i.p.: Intraperitoneal
MD: Molecular dynamics
mCRPC: Metastatic castration–resistant prostate cancer
NASH: Non–alcoholic steatohepatitis
NSG: NOD scid gamma
PCa: Prostate cancer
PR: Progesterone receptor
PSA: Prostate–specific antigen
PSMA: Prostate–specific membrane antigen
RXRα: Retinoid X receptor alpha
RXRβ: Retinoid X receptor beta
s.c.: Subcutaneous
SNVs: Single nucleotide variants
T3: Triiodothyronine
TH: Thyroid hormone
TGI: Tumor growth inhibition
TRβ: Thyroid hormone receptor beta
TSH: Thyroid–stimulating hormone
