# Supplementary material for: Thyroid hormone receptor beta signaling is a targetable driver of prostate cancer growth
Source: Mol Cancer. 2025 Oct 14;24:256. doi: 10.1186/s12943-025-02451-2 (PMC12523147; doi:10.1186/s12943-025-02451-2)
Supplement: Supplementary file 1 — Supplementary Material 1. [file 12943_2025_2451_MOESM1_ESM.docx]

**
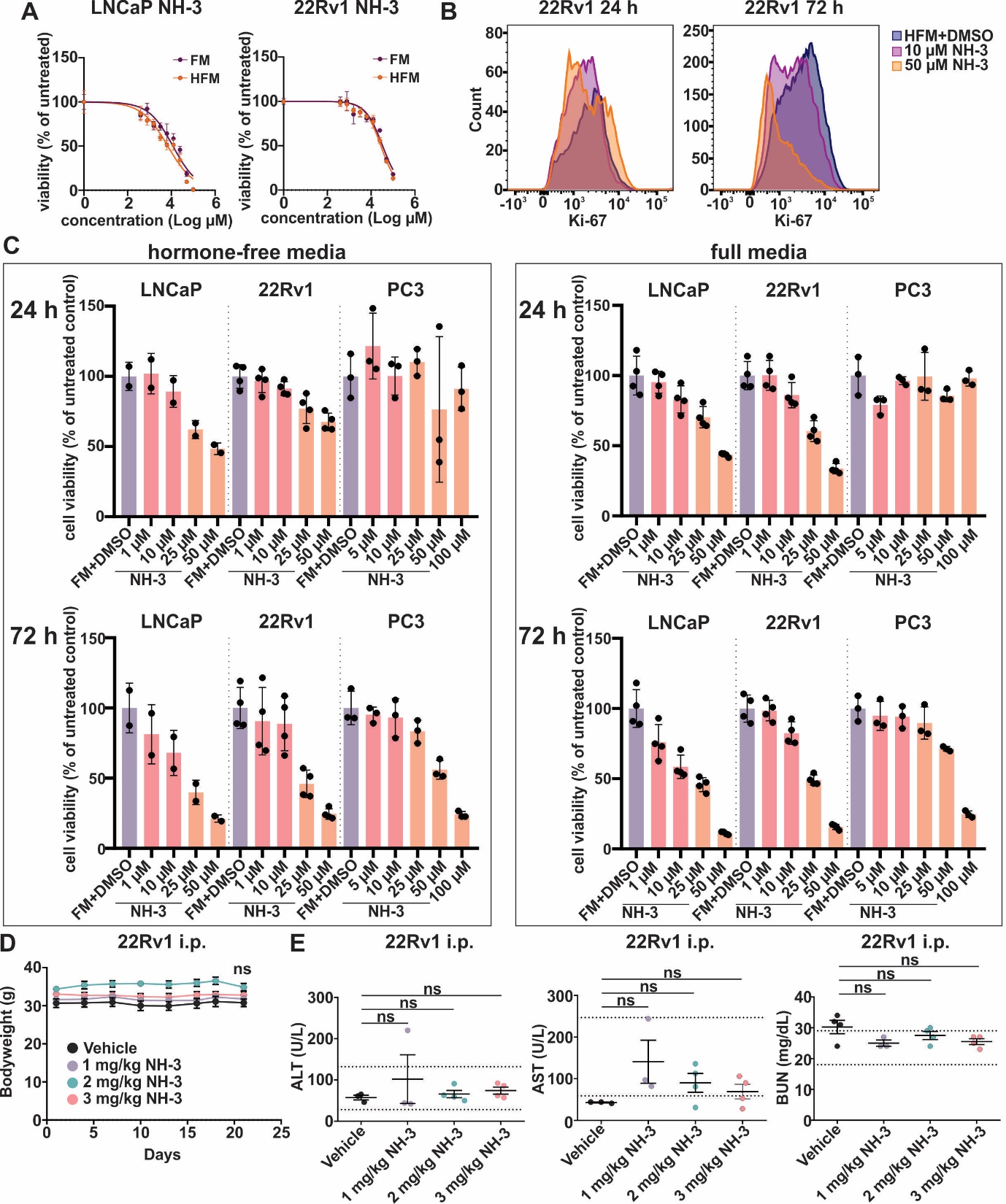
**

**Supplementary Figure 1. (A)** Dose-response curves of NH-3 treated LNCaP and 22Rv1 cells after 72h treatment. **(B)** Reduced proliferation of 22Rv1 cells induced by increasing NH-3 treatment, as seen by a shift towards Ki-67-low expressing cells. **(C**, left panel**)** Reduced proliferation of NH-3 treated LNCaP, 22Rv1, and PC3 in Resazurin assay in hormone-free conditions and **(C**, right panel) in full media. Values were normalized to the mean of untreated controls for each cell line and time point and presented as percentages. **(D)** No alterations of body weight in 22Rv1 xenograft mice upon 1, 2, 3 mg/kg NH-3 treatment compared to controls. **(E)** Unchanged liver and kidney parameters in 22Rv1 xenograft mice upon 1, 2, 3 mg/kg NH-3 treatment compared to controls. Mean ± SD, *p < 0.05, **p < 0.01, and ***p < 0.001. ALT - alanine aminotransferase, AST - aspartate aminotransferase, BUN - blood urea nitrogen.

**
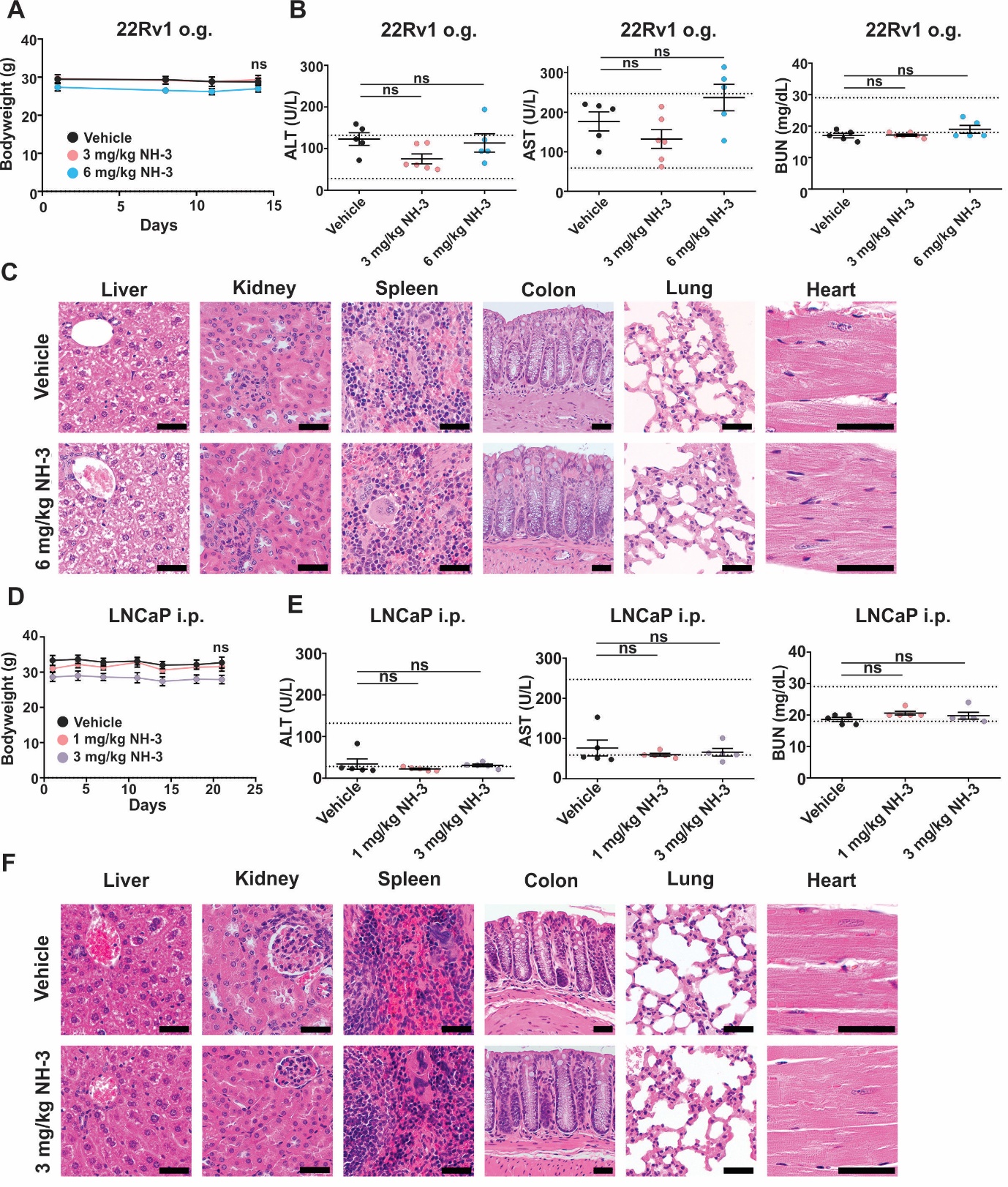
**

**Supplementary Figure 2. (A)** Body weight of 22Rv1 xenograft mice treated with vehicle, 3 and 6 mg NH-3/kg/day by o.g. administration **(B)** Liver and kidney parameters in sera of 22Rv1 xenograft mice treated with 3 and 6 mg NH-3/kg/day by o.g. administration **(C)** Representative H&E images of liver, kidney, spleen, colon, lung and heart of 22Rv1 xenograft mice treated with vehicle and 6 mg NH-3/kg/day by o.g. administration; bars represent 40 µm. **(D)** Body weight of LNCaP xenograft mice treated with vehicle, 1, and 3 mg NH-3/kg/day by i.p. administration. **(E)** Liver and kidney parameters in sera of LNCaP xenograft mice treated with vehicle, 1, and 3 mg NH-3/kg/day by o.g. Mean ± SD, *p < 0.05, **p < 0.01, and ***p < 0.001. ALT - alanine aminotransferase, AST - aspartate aminotransferase, BUN - blood urea nitrogen. **(F)** Representative H&E images of liver, kidney, spleen, colon, lung, and heart of LNCaP xenograft mice treated with vehicle and 3 mg NH-3/kg/day by i.p; bars represent 40 µm.

**
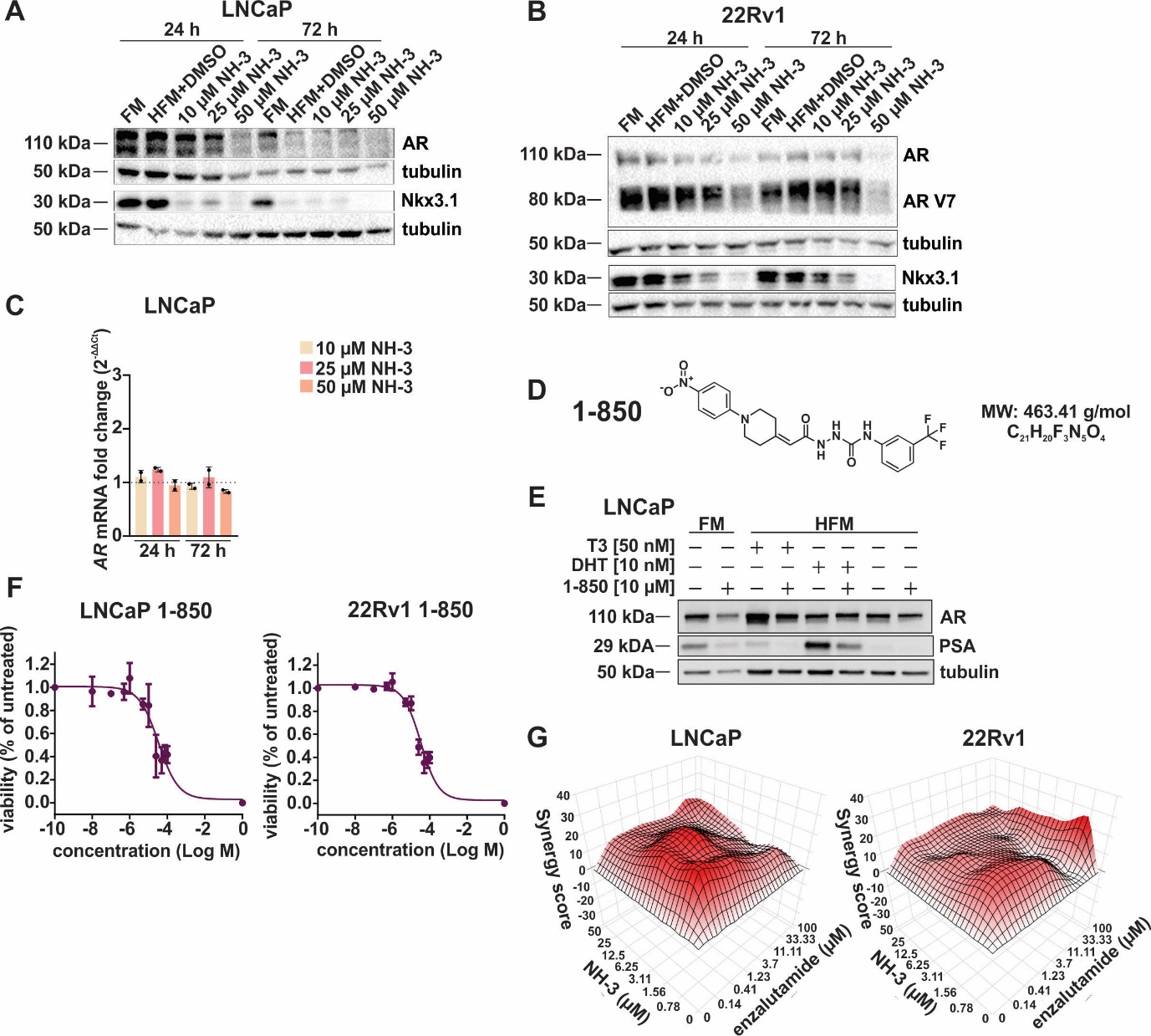
**

**Suppl. Fig. 3a. (A)** Western blot analysis of LNCaP cells treated with increasing NH-3 concentrations after 24 h. Representative blot showing expression of TRβ, AR, PSA, Nkx3.1; GAPDH and tubulin as loading controls. **(B)** Western blot analysis of 22Rv1 cells treated with increasing NH-3 concentrations after 24 h. Representative protein expression of TRβ, AR, PSA, Nkx3.1; GAPDH and tubulin as loading controls. **(C)** AR mRNA levels in LNCaP cells upon NH-3 treatment after 24 and 72 h. Results presented as fold change normalized to β-tubulin. **(D)** Molecular structure of 1-850. **(E)** Western blot analysis of AR and PSA protein expression analyzed in LNCaP cells treated with 10 μM 1-850 in FM, HFM, or HFM supplemented with 1 nM T3 after 72 hours. β-tubulin was used as a loading control. **(F)** IC-50 values for 1-850 in different LNCaP and 22Rv1 cell lines. **(G)** Synergy maps of NH-3, enzalutamide, and combined treatment, created with “Synergy Finder”.

**
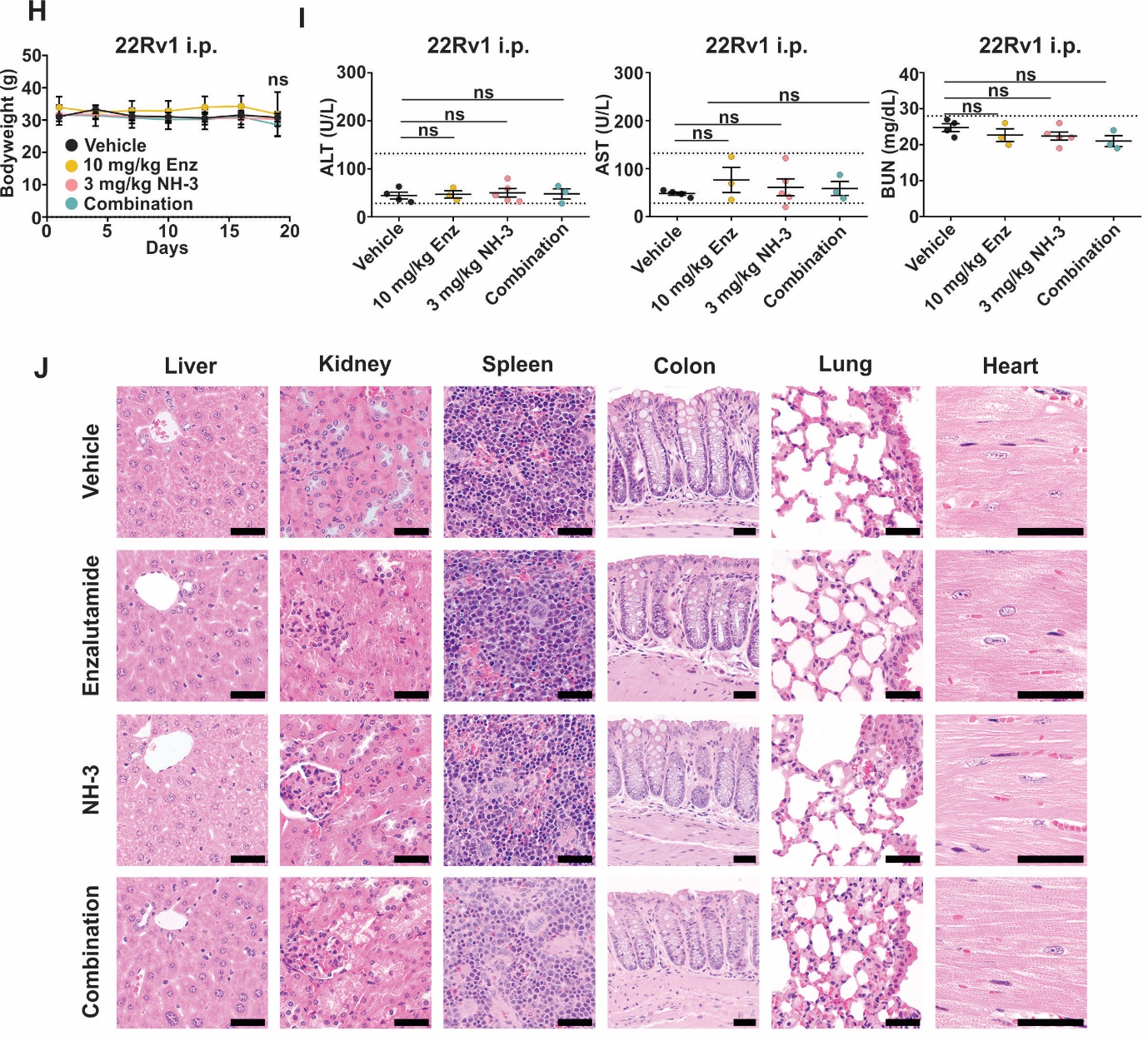
**

**Suppl. Fig. 3b. (H)** Body weights of 22Rv1 xenograft mice upon 3 mg/kg/day NH-3, 10 mg/kg/day enzalutamide and combinatory i.p. treatment. **(I)** Liver and kidney parameters in 22Rv1 xenograft mice upon i.p. 3 mg/kg/day NH-3, 10 mg/kg/day enzalutamide, and combinatory treatment. Mean ± SD, *p < 0.05, **p < 0.01, and ***p < 0.001. ALT - alanine aminotransferase, AST - aspartate aminotransferase, BUN - blood urea nitrogen. (**J**) Representative H&E images of liver, kidney, spleen, colon, lung, and heart of 22Rv1 xenograft mice upon i.p. 3 mg/kg/day NH-3, 10 mg/kg/day enzalutamide, and combinatory treatment; bars represent 40 µm.


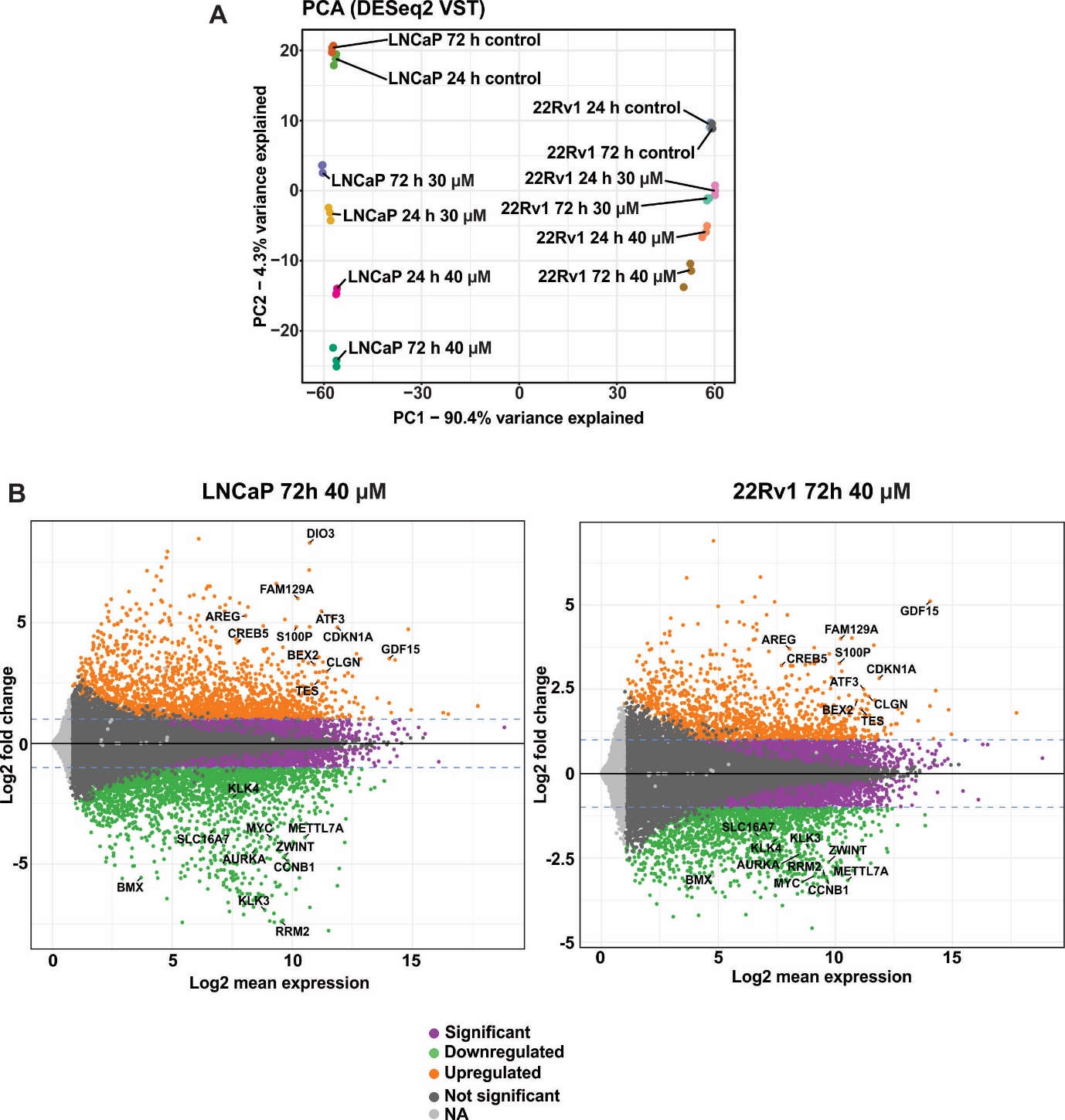


**Suppl. Fig. 4a. (A)** PCA plot of LNCaP and 22Rv1 showing grouping according to NH-3 treatment conditions. **(B)** MA plot showing differentially expressed genes after 40μM NH-3 treatment for 72h across LNCaP and 22Rv1 cells. Genes are colored: in light grey if adjusted p-value (padj) was not reported in DESeq2 results, in dark grey if padj ≥ 0.05, in purple if padj < 0.05 and |log2FC| < log2(1.5), in orange if padj < 0.05 and log2FC > log2(1.5), and in green if padj < 0.05 and log2FC < log2(1.5). Genes of interest are labelled.

**
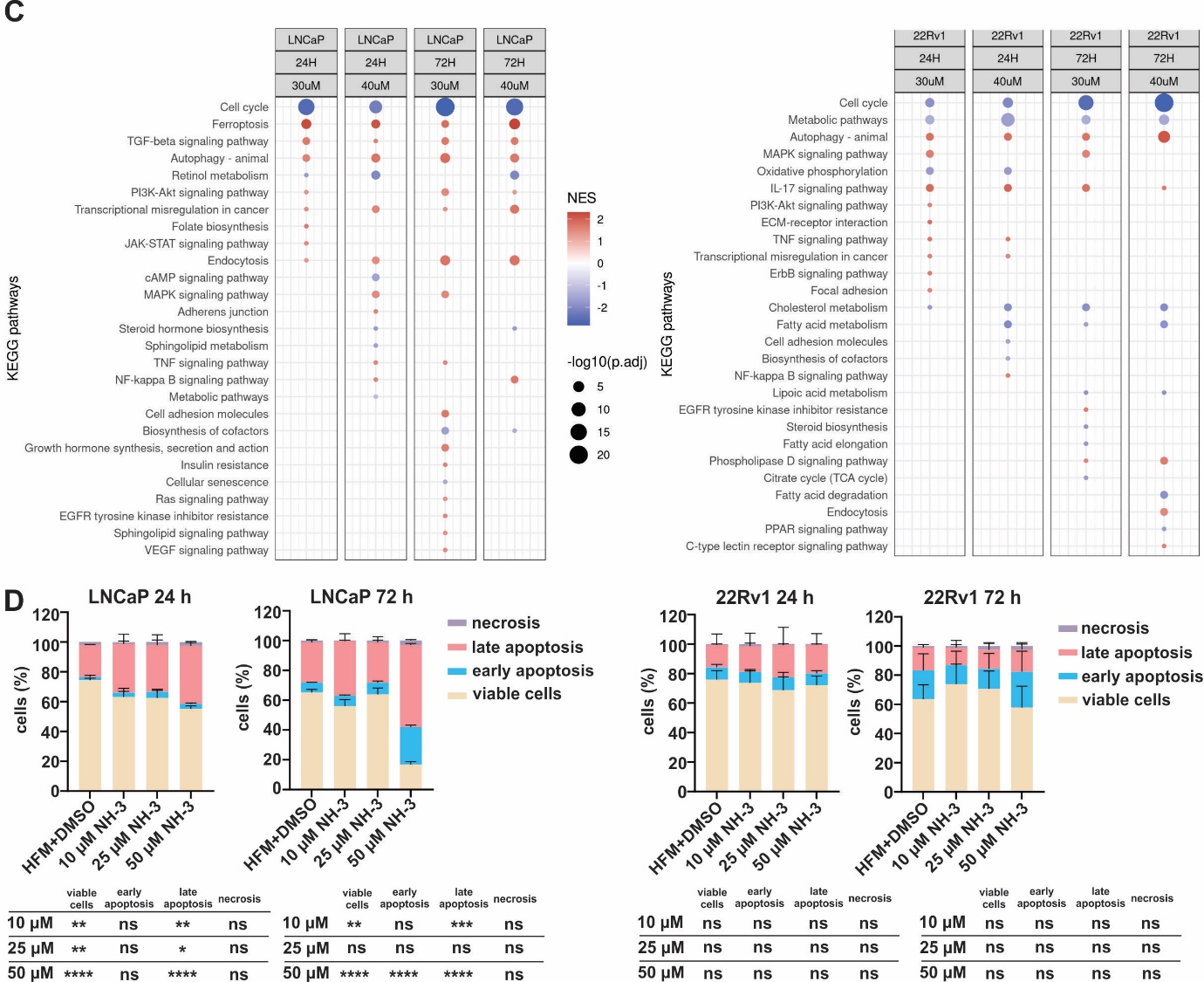
**

**Suppl. Fig. 4b. (C)** Gene set enrichment analysis results of KEGG pathways, showing the most dysregulated pathways in LNCaP and 22Rv1 cells post NH-3 treatment. The size of the bubble corresponds to -log10(adjusted p-value). Red represents upregulated and blue downregulated pathways. **(D)** Enhanced apoptosis of LNCaP but not 22Rv1 cells post NH-3 treatment measured by Annexin V assay. Mean ± SD, *p < 0.05, **p < 0.01, and ***p < 0.001.

**
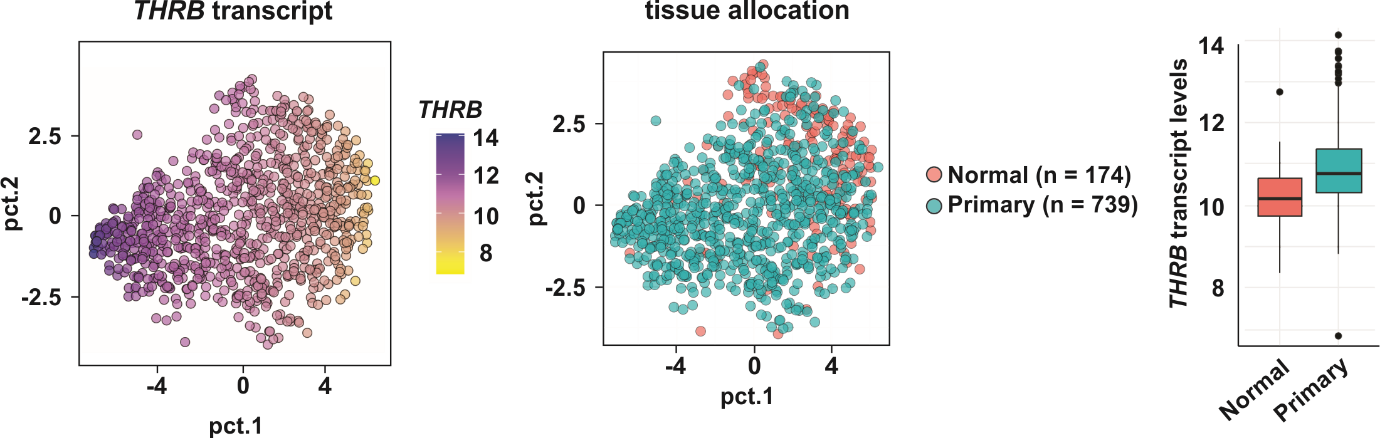
**

**Suppl. Fig. 5.** PCA representation of expression levels of THRB in subsets of primary PCa (Primary, n=739) and normal prostate tissue (NORMAL, n=174), left panels and box plot representation, right panel.
